# The global effect of aortic coarctation on carotid and renal pulsatile hemodynamics

**DOI:** 10.1371/journal.pone.0310793

**Published:** 2024-12-17

**Authors:** Deniz Rafiei, Niema M. Pahlevan

**Affiliations:** 1 Department of Aerospace and Mechanical Engineering, University of Southern California, Los Angeles, California, United States of America; 2 Division of Cardiovascular Medicine, Keck School of Medicine, University of Southern California, Los Angeles, California, United States of America; Chongqing University Three Gorges Hospital, CHINA

## Abstract

Coarctation of the aorta (CoA) is a congenital disease characterized by the narrowing of the aorta, typically the descending portion after the left subclavian artery. If left untreated, by the time individuals reach 50 years of age, the mortality rate can reach 90%. Previous studies have highlighted the adverse effects of CoA on local hemodynamics. However, no study has investigated the global hemodynamic effects of CoA in end-organ (brain and kidney) damage. Clinical studies have shown that coarctation acts as a reflection site, potentially damaging the hemodynamics of the brain and kidneys. Our goal in this study is to investigate the underlying mechanisms of these altered wave dynamics and their impacts on the pulsatile hemodynamics of end-organs. In this study, we use a physiologically accurate in-vitro experimental setup that simulates the hemodynamics of systemic circulation. Experiments are conducted across various cardiac outputs, heart rates, and coarctation degrees using aortas across a wide range of aortic stiffnesses. Our principal finding is that CoA increases cerebral blood flow and harmful pulsatile energy transmission to the brain. Conversely, both renal blood flow and pulsatile energy transmission to the kidneys are reduced in CoA at every level of aortic stiffness.

## Introduction

Coarctation of the Aorta (CoA), the 5th most common congenital heart disease (CHD) [1], is characterized by the narrowing of the aorta (mostly the descending aorta) after the left subclavian artery. Without treatment, 60% of adults over the age of 40 develop heart failure (HF), 75% of patients die by the age of 50, and 90% die by the age of 60 [2]. As aortic coarctation creates a wave reflection site due to an impedance mismatch caused by the narrowing, the early-systolic forward compression wave reflects partially at the narrowed area [3–6]. This wave reflection can still exist after surgical repair because of the possibility of re-coarctation, residual coarctation, local stiffening, or abnormalities in shape [7–11]. A better understanding of the pulsatile hemodynamic changes caused by altered wave reflection in aortic coarctation patients can help improve the management and treatment of CoA patients.

Several studies have investigated the effect of aortic coarctation on arterial wave reflections and their hemodynamic consequences [3–6]. Mynard et al. performed a detailed study on how altered wave reflection in CoA affects cerebral hemodynamics and left ventricular load, using a sheep animal model and a validated hybrid 0D-1D model of the entire adult circulation [12]. The local hemodynamic effects of aortic coarctation, including wall shear stress and local pressure drop, have also been explored by several groups [13–15]. Complementary to previous work, our study uses a physiologically accurate in-vitro setup of the coupled atrioventricular-aortic system [16, 17] to investigate the effect of altered wave reflections on pulsatile hemodynamics of renal and cerebral systems, potentially leading to end-organ damage. In particular, we have focused on pulsatile energy transmission and volume blood flow toward end organs (brain and kidney). It is well-accepted that the pulsatile nature of central hemodynamics has a harmful effect on vital organs. High pulsatile power can exacerbate end-organ damage, particularly in organs like the brain and kidneys, which are characterized by high blood flow and low resistance [18–20]. Clinical studies have shown that elevated pulsatile power in the brain is associated with cognitive impairment and damage to small cerebral vessels [21, 22]. Furthermore, it is well-established that decreased renal blood flow pulsatility (i.e., reduced pulsatile hemodynamic energy) significantly impacts kidney function, potentially leading to acute renal insufficiency or failure as a result of increased renin secretion [23]. Therefore, incorporating pulsatile power into our analysis offers a deeper understanding of how aortic coarctation may contribute to the damage of these vital organs.

To the best of our knowledge, the global effects of CoA on the pulsatile hemodynamics of cerebral and renal arteries (specifically in terms of pulsatile energy transmission and blood flow distribution) and the extent of these hemodynamic alterations have not been thoroughly investigated. This could be mainly due to numerous confounding variables that cannot be studied on patients, such as heart rate (HR), cardiac output (CO), aortic compliance, and the degree of coarctation. Our unique atrioventricular-aortic in-vitro simulator [16, 17] enabled us to simulate various physiological conditions and perform detailed quantification of hemodynamics, including pressure and flow waveforms at different locations, which are crucial for understanding the potential damage to end organs caused by CoA. These types of variations and detailed measurements are not feasible in clinical studies due to concerns for patient safety and the associated costs. Additionally, this setup allowed us to study CoA’s differential effects within a controlled environment, enabling the focused study of a single variable while keeping all others constant.

Aortic coarctation can manifest in simple or complex forms [24, 25] and sometimes presents with heart failure [26]. Recognizing the significance of its clinical variations, this study extends beyond the conventional focus on non-heart failure patients and includes experiments under low cardiac contractility (e.g., low cardiac output) to simulate CoA patients with heart failure, aiming to comprehensively assess the effects of coarctation on both brain and kidney function across the clinical spectrum of patient groups. The findings of the present study serve as an essential first step toward understanding the hemodynamic mechanisms of aortic coarctation in end-organ damage. They also can potentially aid in developing targeted patient-specific therapeutic approaches for these patients through a better understanding of these mechanisms.

## Materials and methods

### In-vitro experimental setup

**Fig 1:**
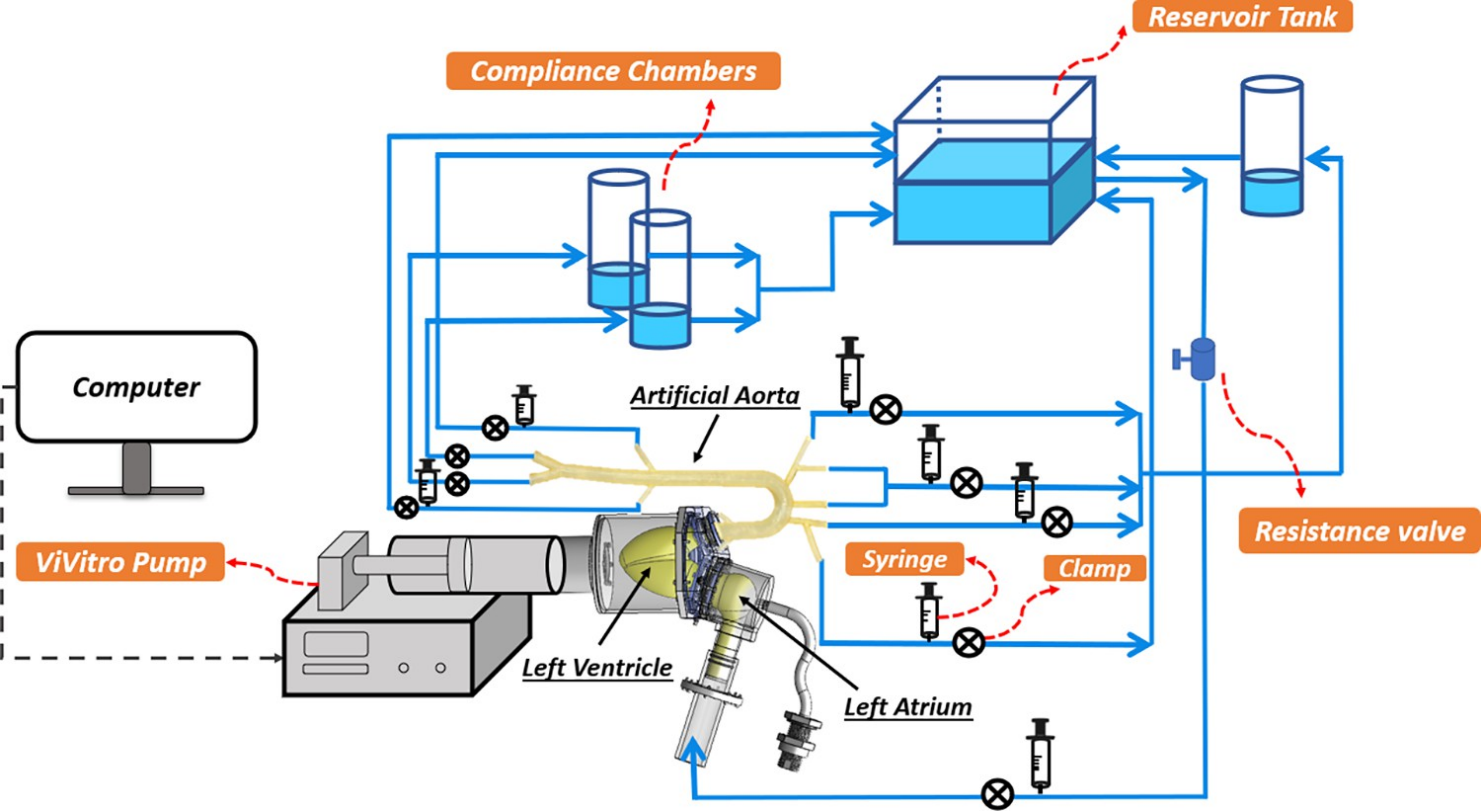
The full schematic of the atrioventricular-aortic experimental setup.

**Fig 2:**
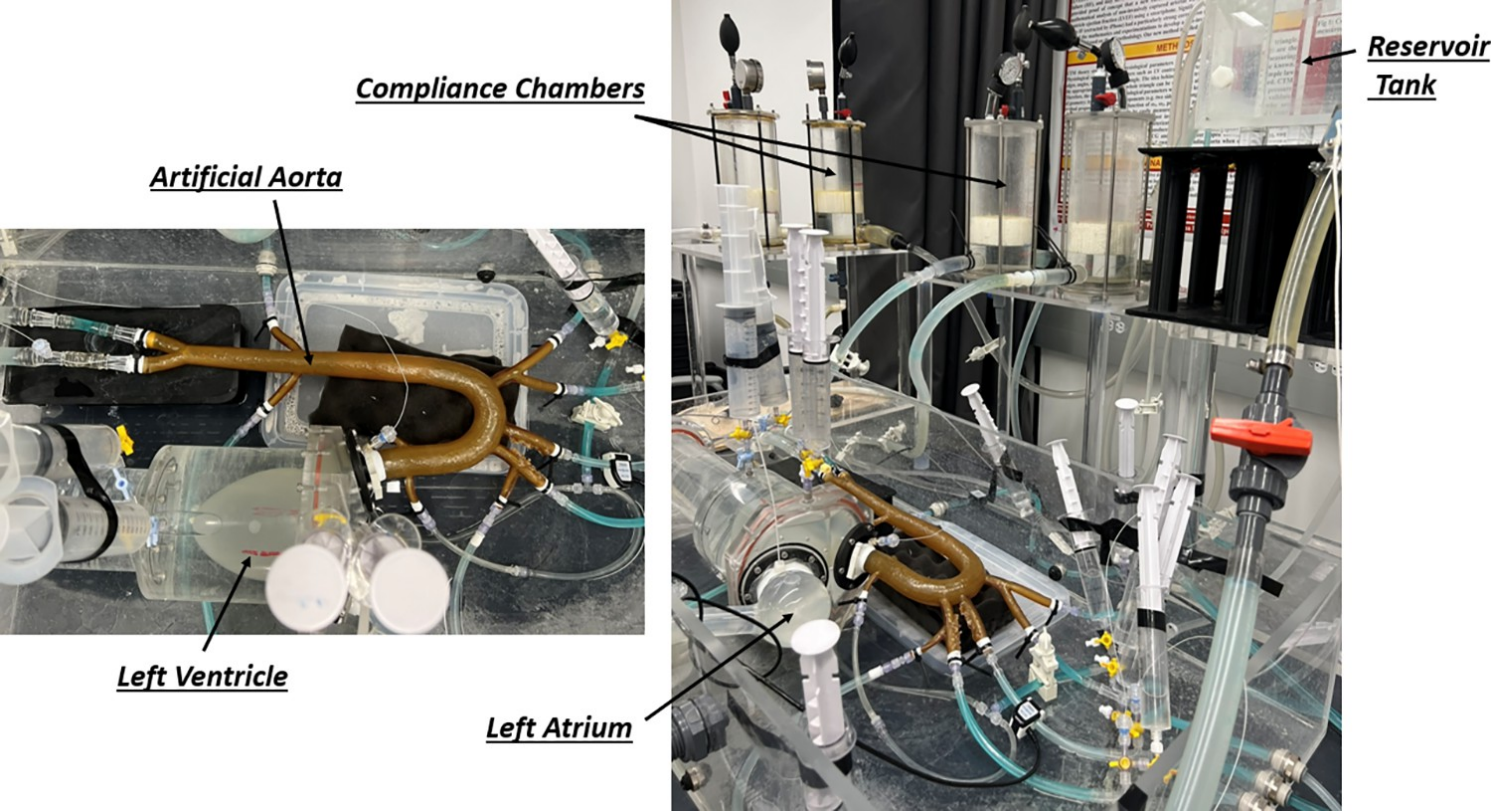
Picture of the in-vitro circulation setup consisting of the LV, LA, the artificial aorta, and the vascular components of the systemic circulation.

Our in-vitro experimental setup comprises two main components: 1) an atrioventricular simulator and 2) an aortic simulator. The full schematic of the system is presented in Fig 1. The atrioventricular simulation system consists of a compliant left ventricle (LV) connected to the aortic root via an artificial aortic valve and on the other side to the artificial left atrium (LA) via an artificial mitral valve (Medtronic MOSAIC1 305 CINCH1). The aortic simulator is composed of the artificial aorta with its main branches (see Fig 1 in S1 File) and end-organ simulators located at the termination of each branch that capture the effects of compliance and resistance of the eliminated vasculature in each branch (see [17, 27] for further details). Three compliance chambers made of acrylic glass are used to simulate the total arterial resistance and compliance of the vascular system. An open tank connected to the left atrium constitutes the venous reservoir. To create a systolic contraction, the compliant LV sac is squeezed inside a fluid-filled plexiglass container using a programmable piston pump (ViVitro Labs Inc, SuperPump, AR SERIES), which is set to simulate specific contractility and contractile motions (ViVitro Labs Inc.). The vivigen interface on the computer unit allows us to adjust the frequency of the pump operation, which determines the heart rate. As it is well established that fluid viscosity has almost no effect on arterial waves [28], water is used as the circulating fluid in all experiments. A picture of the final fabricated setup is shown in Fig 2.

### Compliant aortic models with coarctation

The aortas used in this study were fabricated using natural latex rubber (Chemionics Corp.) and silicone rubber (RTV-3040, Freeman Manufacturing & Supply Company). These materials have mechanical characteristics (relevant to hemodynamics studies) that are similar to those of natural human aortas [17]. Aortic phantom fabrication process involves the following steps: For latex aorta: a stainless-steel metal aortic mold is dipped into liquid latex followed by curing the coated material at standard room temperature (25˚C) for two hours. If necessary, additional coating layers were applied to achieve the desired aortic compliance. For silicone aorta: the base of the silicon rubber (RTV-3040, Freeman Manufacturing & Supply Company) and the catalyst were mixed; then we coated a light silicon sheet of 25 g of the mixed solution. We let the coated material for 16 hours at standard room temperature (25˚C); finally, we repeated the coating for more layers (as required) to achieve the desired aortic compliance. The fabrication process details can be found in the S1 File. For each aorta, the aortic stiffness is evaluated by measuring the pulse wave velocity (PWV) using the foot-to-foot method. This measurement technique involves determining the delay time between the first and second propagating waves at the aortic root and femoral bifurcation, respectively. Aortic compliance (AC) is determined for each aorta by adding small amounts of fluid to the system and measuring the increase in pressure [16, 17]. Table 1 displays the PWV and AC measurements of the fabricated aortas used in the study.

**Table 1:** Properties of the fabricated aortas.

Aorta No.	Material	PWV (m/s)	AC (mL/mmHg)
Aorta-1	Silicone	8	1.2
Aorta-2	Silicone	10.5	1.15
Aorta-3	Latex	19	1
Aorta-4	Silicone	22.5	0.75
Aorta-5	Latex	24	0.6
Aorta-6	Latex	28	0.35

The coarctation degree was defined as the following: $100\%\times (1-{Area}_{Coarctation}/{Area}_{Descending})$ [14]. To model aortic coarctation, a zip tie was used on the descending aorta 6 cm after the left subclavian artery. Three different coarctation degrees were implemented by adjusting the zip tie for each aorta: mild (25% narrowing of the cross-sectional area), moderate (50% narrowing), and severe (75% narrowing).

### Measurements

Pressure waveforms are measured by a Millar MIKRO-TIP1 Catheter Transducer using a PowerLab 4/35 from AD Instruments. Measurement sites are chosen as the following: The ascending aorta (5 cm apart from the aortic root), central LV, central LA, at the bifurcation, in right renal (30 mm from the inlet), and in the middle (30 mm from the inlet) of the left common carotid artery. Flow is also measured by a Transonic Flowmeter (TS410) in ascending aorta (Clamp-on ME20PXL flow sensor), left common carotid artery (Clamp-on ME6PXL flow sensor), and renal artery (Inline ME6PXN flow sensor). These simultaneously measured flow and pressure waveforms are used to quantify pulsatile energy transmission and to perform wave analysis, as detailed in the next section.

### Hemodynamic analysis

#### Pulsatile power

Pulsatile power has been investigated by several groups as a well-established method for understating the wave dynamics [29–31]. The pulsatile power transmitted to the brain and kidneys (*P*_*pulse*_) is calculated using the following formula:

$$P_{pulse}=\frac{1}{T}\int_{0}^{T}p(t)q(t)dt-\frac{1}{T^{2}}\int_{0}^{T}p(t)dt\int_{0}^{T}q(t)dt$$ (1)

Where T represents the cardiac cycle period, and *p*(*t*) and *q*(*t*) are the pressure and flow waveforms, respectively.

#### Wave intensity

The well-established wave intensity (WI) analysis [32] was performed in cerebral and renal arteries using pressure and velocity measurements simultaneously. Wave intensity (*dI*) is defined as the product of the pressure changes and velocity changes during a small-time interval, which is calculated using Eq 2:

dI=dP.dU (2)

Here, *dP* and *dU* represent changes in pressure and velocity, respectively. These increments of pressure and velocity are divided by the time interval (*dP*/*dt* and *U*/*dt*) to remove the reliance of *dI* on the sampling frequency (time steps). Therefore, net wave intensity is shown in the units of power per unit area per unit time (*W*.*s*^−2^.*m*^−2^) [32].

#### Power spectra

Power spectra are obtained using the Fast Fourier Transform (FFT) [21]. They represent the distribution of signal energy throughout different frequencies. In the present study, the power spectra of blood flow at the carotid and renal arteries are calculated using Eq 3.


$$S(f)=\frac{1}{L}{\left|FFT(x(n))\right|}^{2}$$ (3)

Where *S*(*f*) and *x*(*n*) and L represent power spectra, the signal, and the total number of samples in the signal, respectively.

### Experimental procedure

To investigate the differential effect of the determinant of the wave dynamics in our CoA models, we repeated experiments under varied physiological and hemodynamic conditions. These included different heart rates (ranging from 45 to 180 bpm); different cardiac outputs (2.5 and 5 L/min) to simulate various levels of heart contractility (normal vs. HF); different aortic stiffnesses (provided in Table 1) as the determinants of wave speeds (which also address vascular age); and various degrees of coarctation (mild, moderate, and severe).

From a physics perspective, like any other wave phenomenon, arterial wave dynamics is dominated by three factors: the fundamental frequency of the waves (i.e., heart rate), wave speed (aortic stiffness), and the location of the reflection site. By varying different aortic stiffness and heart rates, we were able to generate different wave dynamics and wave conditions [33]. from the physiological standpoint, we aimed to use aortas with different stiffnesses corresponding to aging [34] and different diseases [35]. Given the natural variability in heart rate among individuals (male/female [36], young/old [37]) and the effects of various medications on the heart rate, investigating the effect of heart rates was needed.

In analyses conducted for this study, each operation point for the measurement was repeated five times. The data point and error bars in the figures are associated with these five measurements.

## Results

### Aortic coarctation and brain pulsatile hemodynamics

#### The effect of coarctation degree on carotid pressure

**Fig 3:**
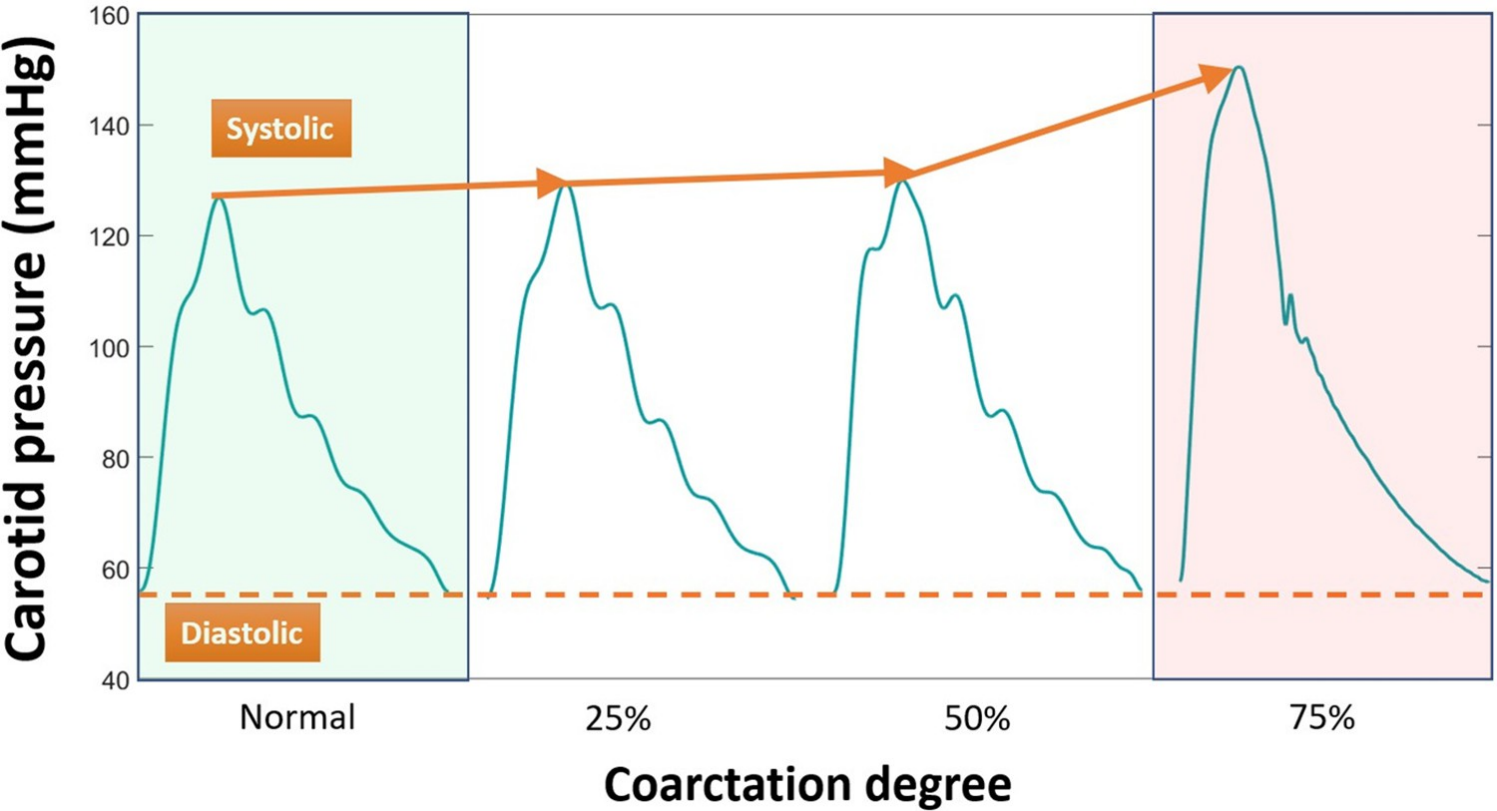
Left common carotid artery pressure waveform before and after coarctation (Aorta-3, CO = 5 L/min, HR = 75bpm).

Fig 3 presents the diastolic, pulse, and systolic pressure of the carotid artery for each coarctation degree for one of the aortas (Aorta- 3). Minimal alterations in diastolic pressure were observed following the imposition of severe coarctation, aligning with the established principle that wave dynamics have negligible impact on diastolic pressure. However, systolic and pulse pressure increase, especially after 50% coarctation. It should be noted that normal, in Fig 3, refers to the aorta without coarctation.

#### The effect of coarctation degree on pulsatile power transmission to the brain

**Fig 4:**
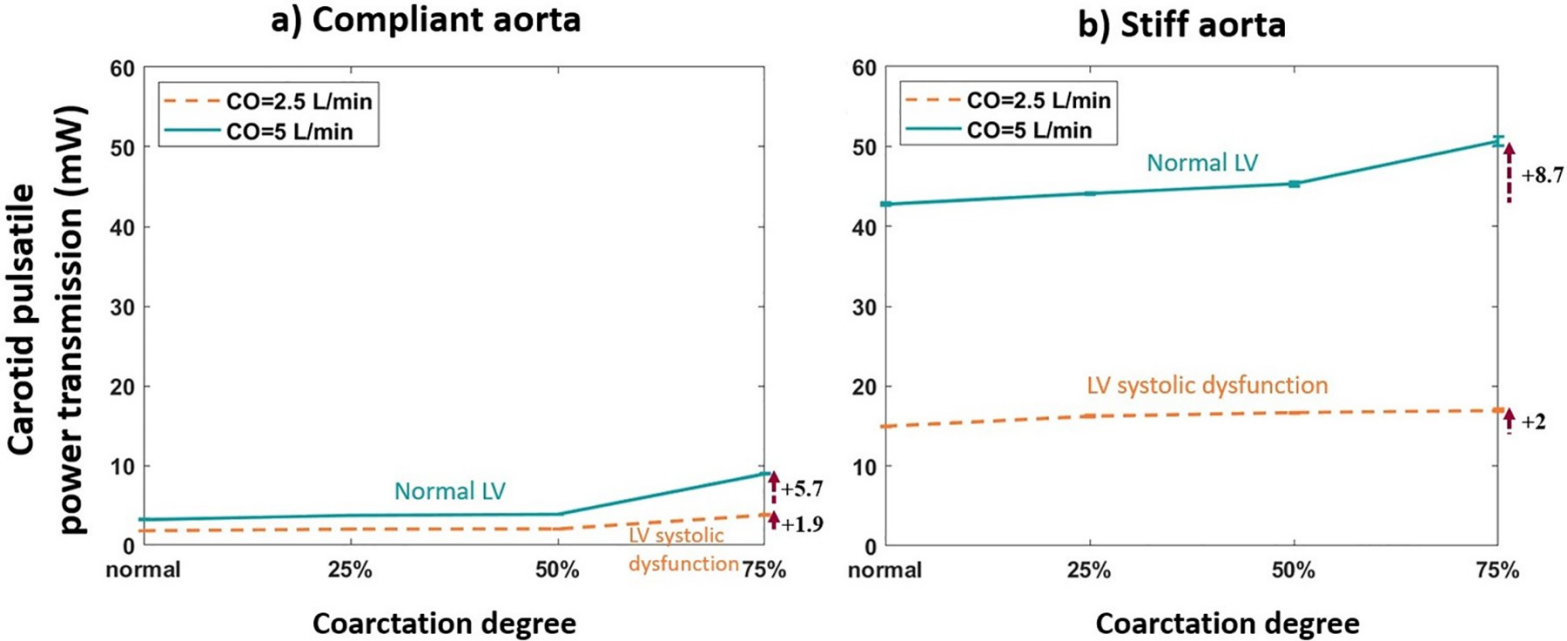
Carotid pulsatile power vs. coarctation degree for the a) compliant and b) stiff aortas (PWV = 8 and 28 m/s; AC = 1.2 and 0.35 mL/mmHg) (HR = 75 bpm). The change is calculated from the normal base.

Fig 4 shows the effect of different coarctation degrees on pulsatile power transmission to the brain. For better visualization, results for the aortas with the highest and lowest compliances (PWV = 8 and 28 m/s; AC = 1.2 and 0.35 mL/mmHg) are shown in this figure (data for other aortas are provided in the S1 File). The blue line represents normal LV function, with a cardiac output (CO) of 5 L/min. The orange dashed line is for LV systolic dysfunction that mimics the heart failure patients, where CO = 2.5 L/min.

#### The effect of coarctation degree on carotid flow

**Fig 5:**
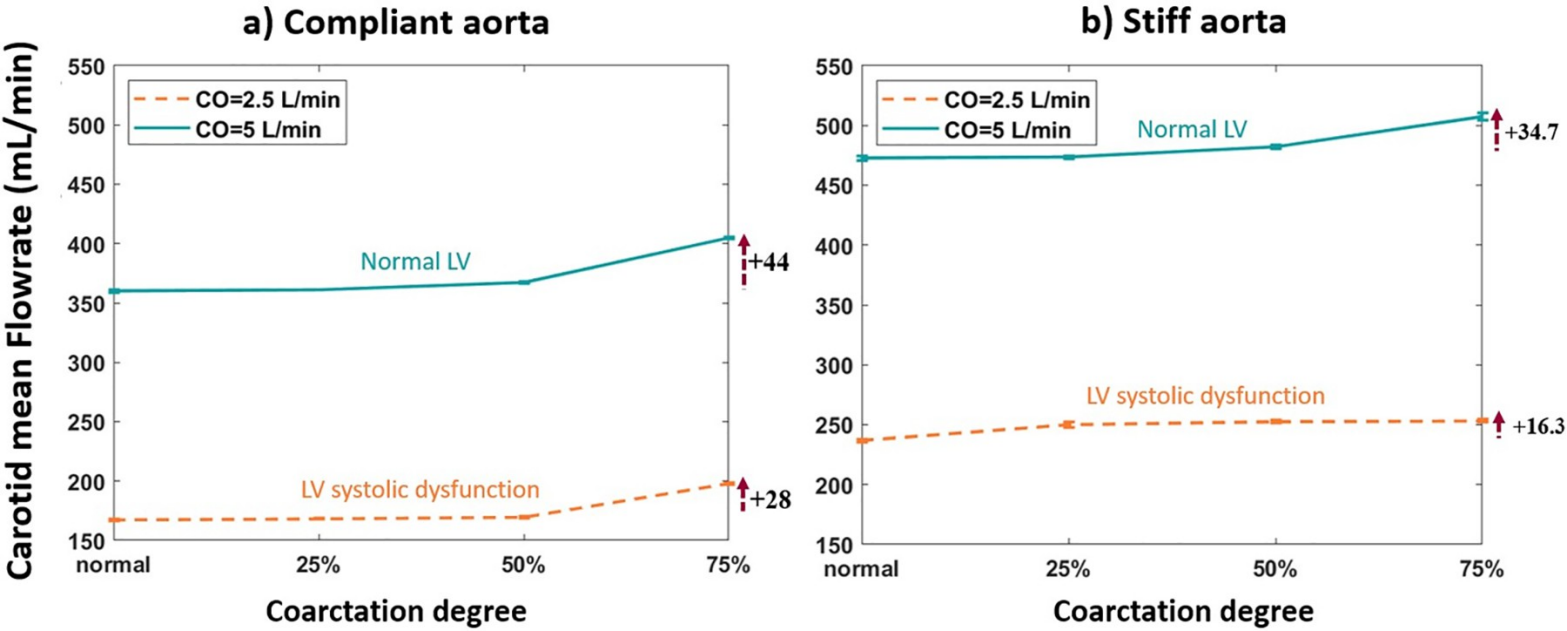
Carotid mean flowrate vs. coarctation degree for the a) compliant and b) stiff aortas (PWV = 8 and 28 m/s; AC = 1.2 and 0.35 mL/mmHg) (HR = 75 bpm).

Fig 5 demonstrates the carotid artery mean volumetric flowrate vs. coarctation degree for the aortas with the highest and lowest compliances (PWV = 8 and 28 m/s; AC = 1.2 and 0.35 mL/mmHg).

#### Variations in brain pulsatile power transmission with coarctation severity for different aortic compliances

**Fig 6:**
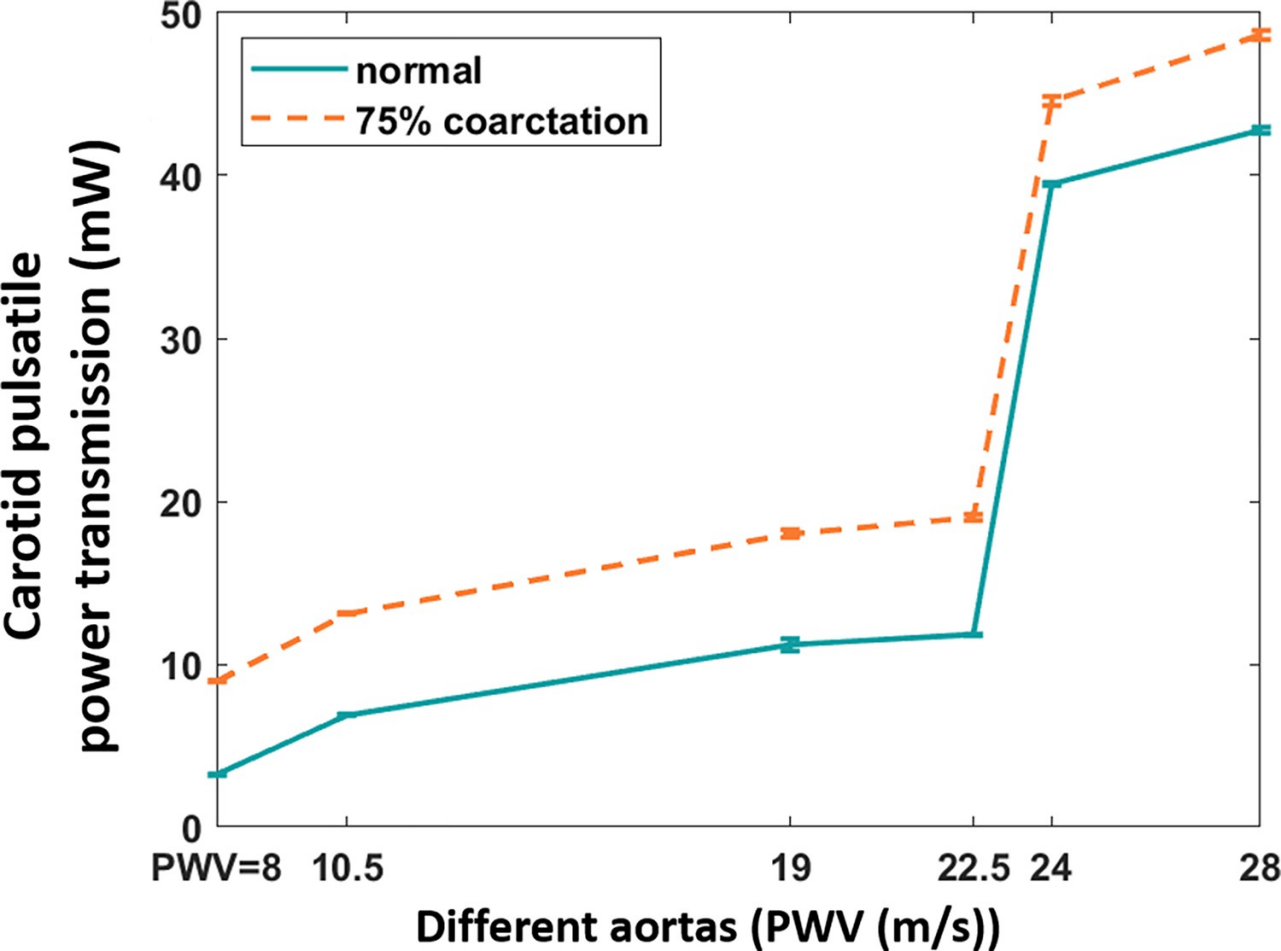
Carotid pulsatile power vs. different aortas for normal and 75% coarctation (CO = 5 L/min, HR = 75 bpm).

Fig 6 represents the Carotid pulsatile power for different aortic rigidities (different ages) under normal and 75% coarctation conditions. As the aorta gets more rigid, energy transmission to the brain increases for both normal and coarctation cases. The addition of coarctation increases the harmful pulsatile power transmission, but the overall pattern of pulsatile transmission and aortic stiffness remains the same.

The figure for carotid pulsatile power vs. coarctation degree for different aortic compliances is also provided in the S1 File.

#### The effect of heart rate on energy transmission to the brain for normal and coarctation cases

**Fig 7:**
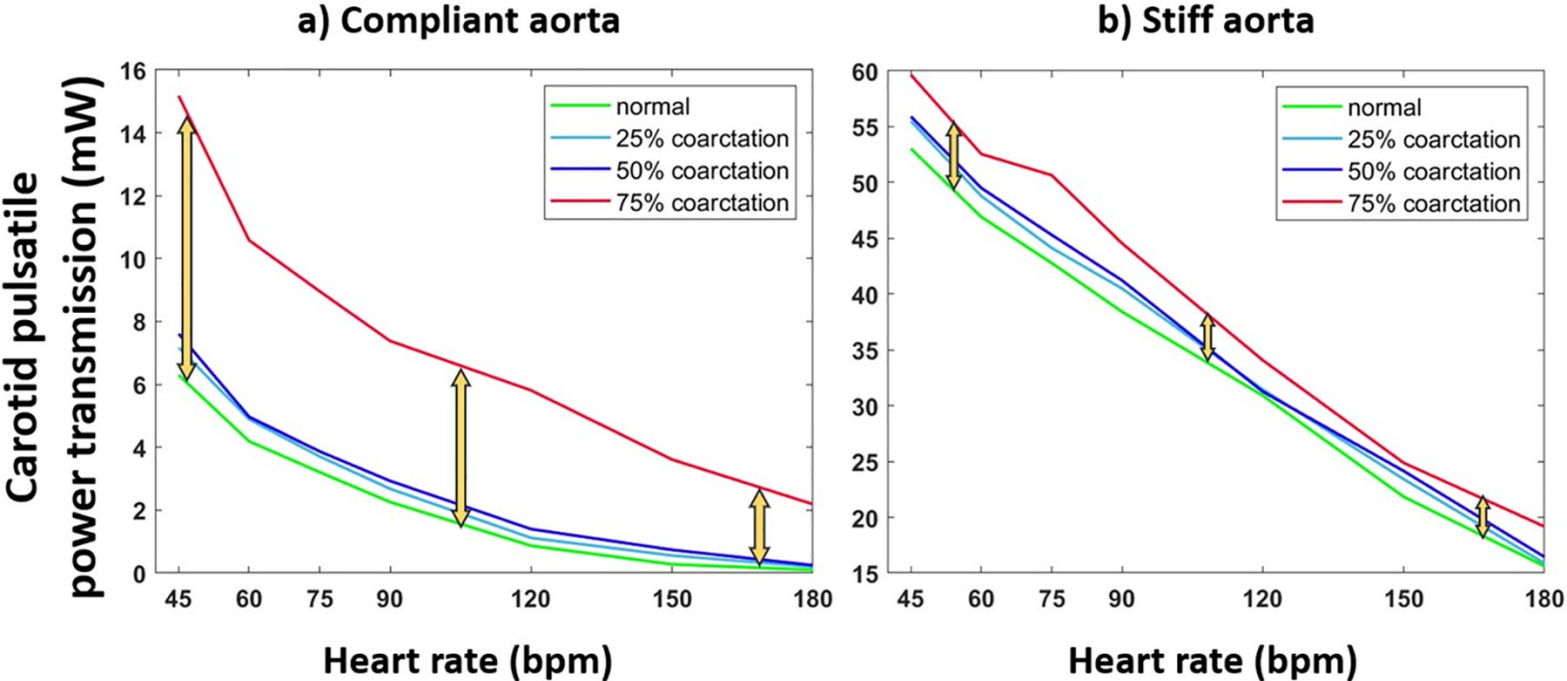
Carotid pulsatile power vs. heart rate for the a) compliant and b) stiff aortas (PWV = 8 and 28 m/s; AC = 1.2 and 0.35 mL/mmHg) under normal and coarctation conditions (CO = 5 L/min, HR = 75 bpm).

Fig 7 demonstrates the energy transmission to the brain changing with heart rate at a fixed CO of 5 L/min for the compliant and stiff aortas under normal and CoA conditions. In our experimental setup, the overall trend shows that as the heart rate increases, energy transmission to the brain decreases for both normal and coarctation conditions. Yellow arrows show the difference in transferred energy between two cases of normal and 75% coarctation.

#### Wave intensity analysis

**Fig 8:**
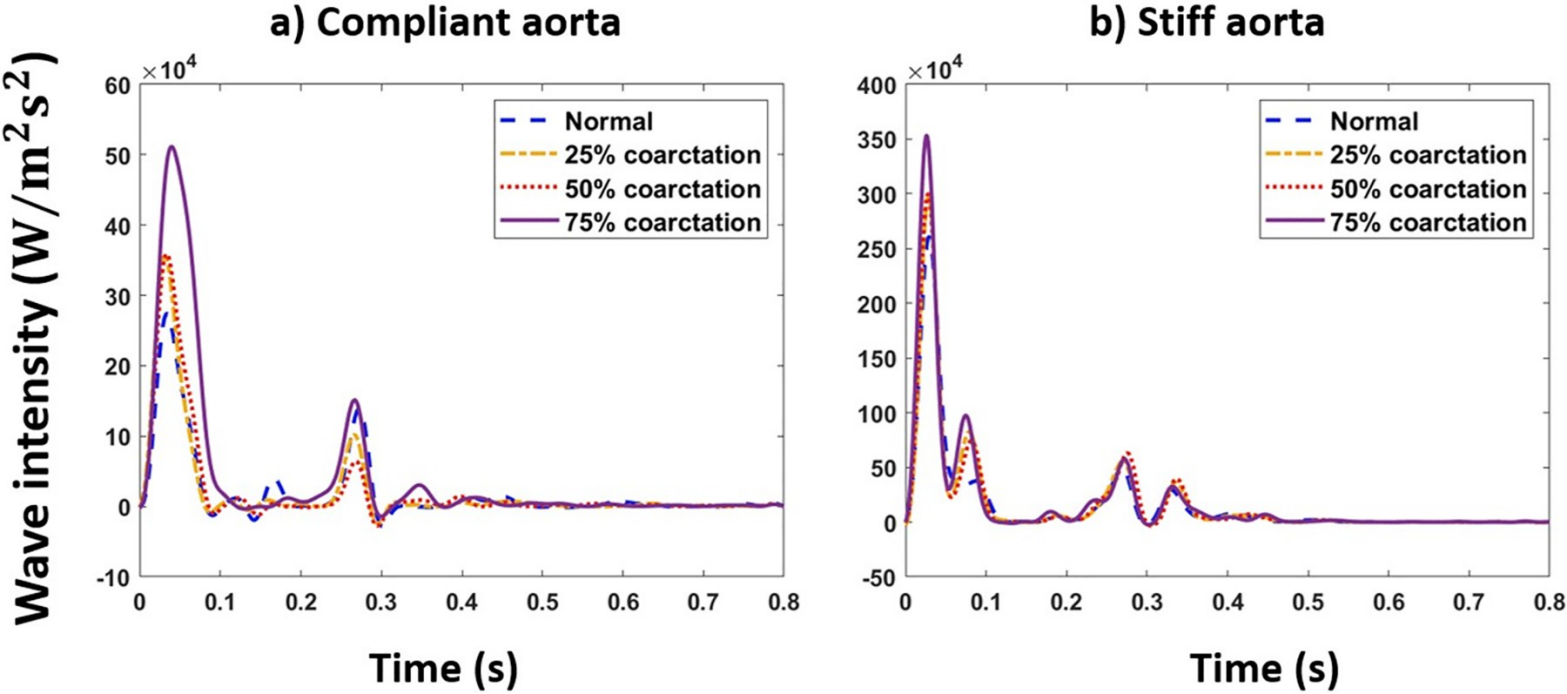
Wave intensity profiles for normal and different coarctation degrees at the carotid artery for a) compliant and b) stiff aortas (PWV = 8 and 28 m/s; AC = 1.2 and 0.35 mL/mmHg) CO = 5 L/min and HR = 75 bpm. Y-axis limit is different for these two figures for better visualization.

Fig 8 shows the total wave intensity profiles for normal and coarctation cases at the carotid artery for the aortas with the highest and lowest compliances (PWV = 8 and 28 m/s; AC = 1.2 and 0.35 mL/mmHg)

**Fig 9:**
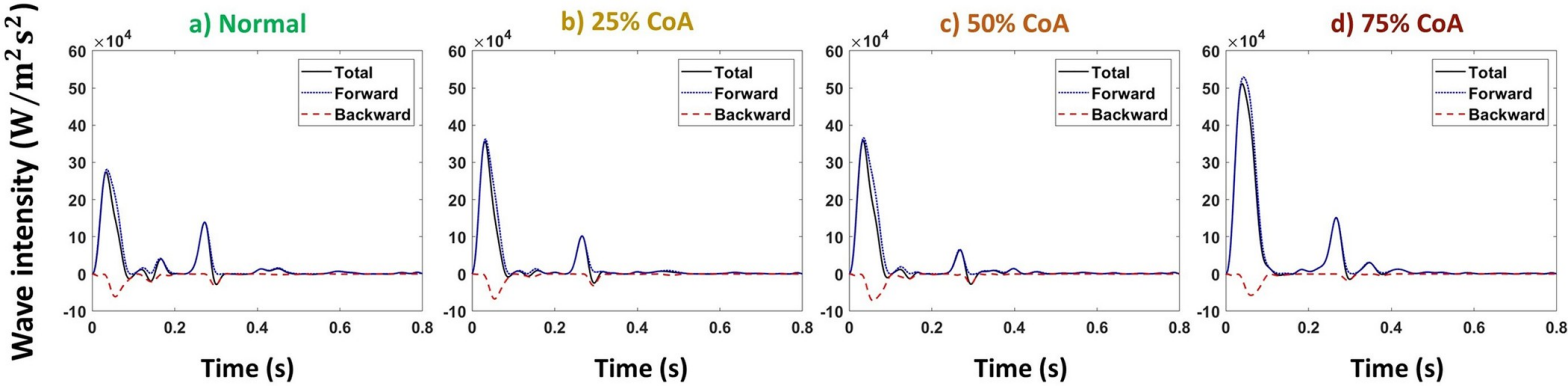
Total, forward, and backward wave intensities at carotid artery for compliant aorta (PWV = 8 m/s, AC = 1.2 mL/mmHg) for a) normal, b) 25%, c) 50%, and d) 75% coarctation.

**Fig 10:**
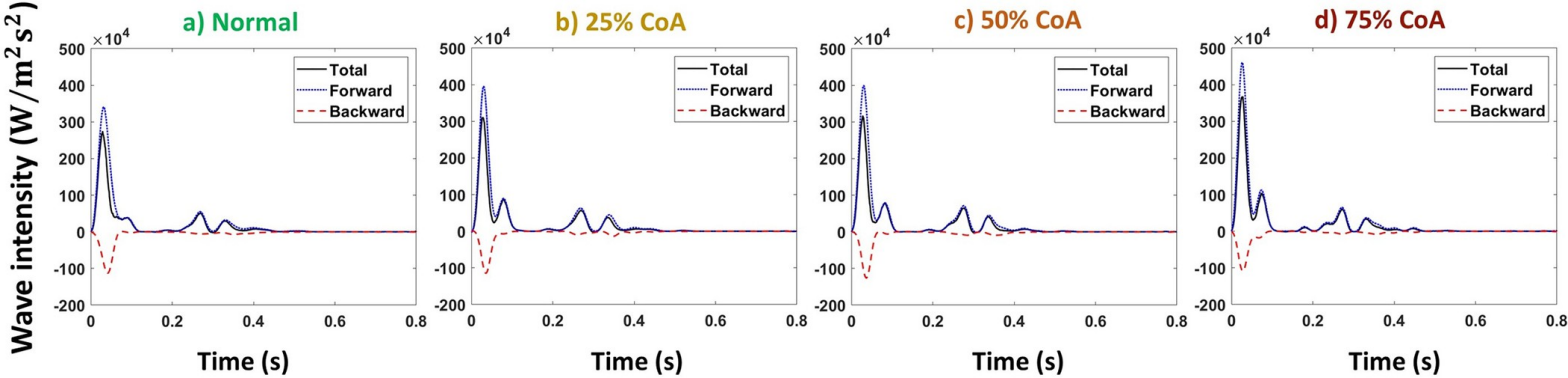
Total, forward, and backward wave intensities at carotid artery for stiff aorta (PWV = 28 m/s, AC = 0.35 mL/mmHg) for a) normal, b) 25%, c) 50%, and d) 75% coarctation.

Figs 9 and 10 represent the total, forward, and backward wave intensity profiles at the carotid artery for normal and coarctation cases for the compliant and stiffened aortas, respectively.

#### Power spectrum analysis

**Fig 11:**
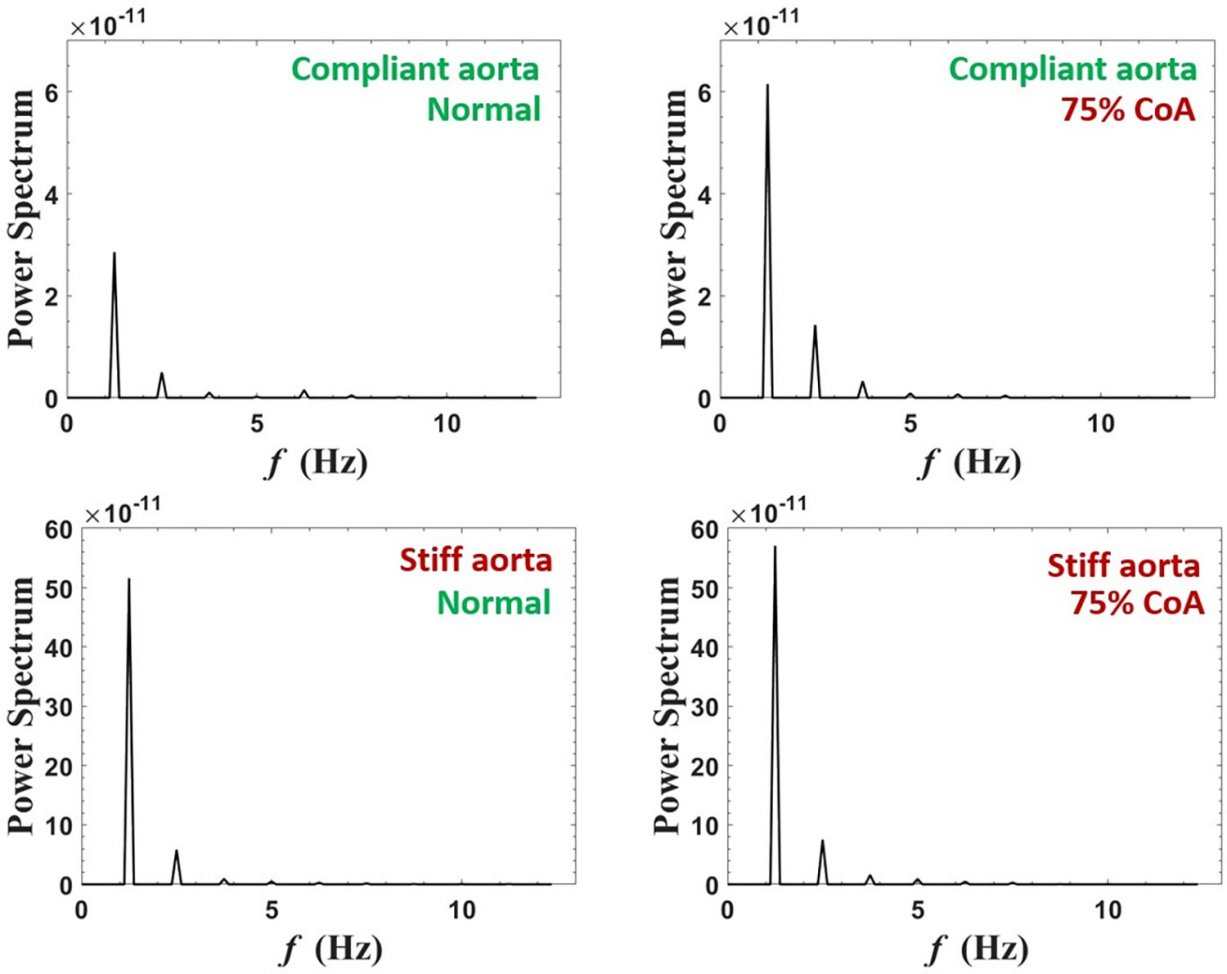
Power spectra of carotid artery flow for normal and 75% coarctation cases for the most compliant and the stiffest aortas (PWV = 8 and 28 m/s; AC = 1.2 and 0.35 mL/mmHg) (CO = 5 L/min, HR = 75 bpm).

Power spectrum analysis of blood flow at the carotid artery is shown in Fig 11 for normal case and 75% coarctation case for the aortas with highest and lowest compliances (PWV = 8 and 28 m/s; AC = 1.2 and 0.35 mL/mmHg). The complete figure that also illustrates 25% and 50% coarctation cases is represented in the S1 File to avoid overcrowding.

### Aortic coarctation and renal pulsatile hemodynamics

#### The effect of coarctation degree on pulsatile power transmission to the kidneys

**Fig 12:**
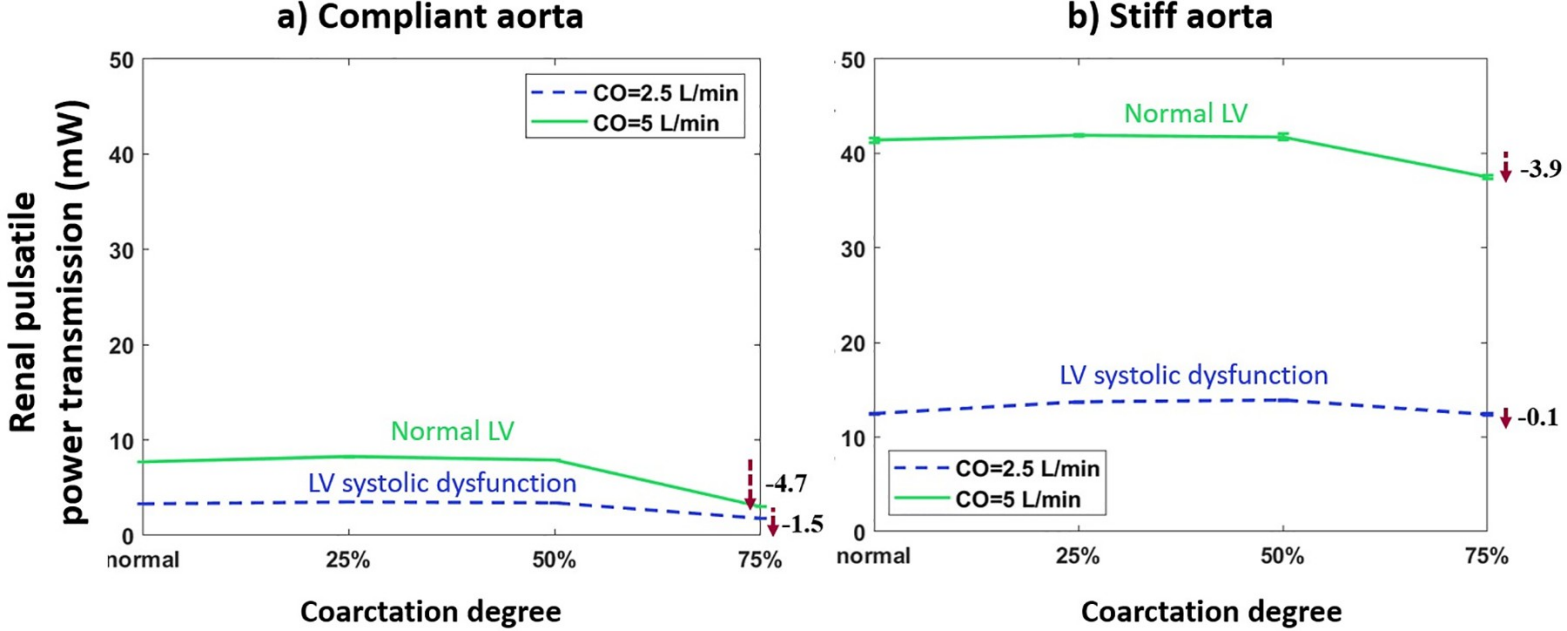
Renal pulsatile power vs. coarctation degree for the a) compliant and b) stiff aortas (PWV = 8 and 28 m/s; AC = 1.2 and 0.35 mL/mmHg) (HR = 75 bpm).

Fig 12 depicts the pulsatile power (energy) transmission to the kidneys vs. coarctation degree for the aortas with the highest and lowest compliances (PWV = 8 and 28 m/s; AC = 1.2 and 0.35 mL/mmHg) (data for other aortas are provided in the S1 File). The green line represents normal LV with CO = 5 L/min, and the dashed blue line represents LV systolic dysfunction with CO = 2.5 L/min.

#### The effect of coarctation degree on renal flow

**Fig 13:**
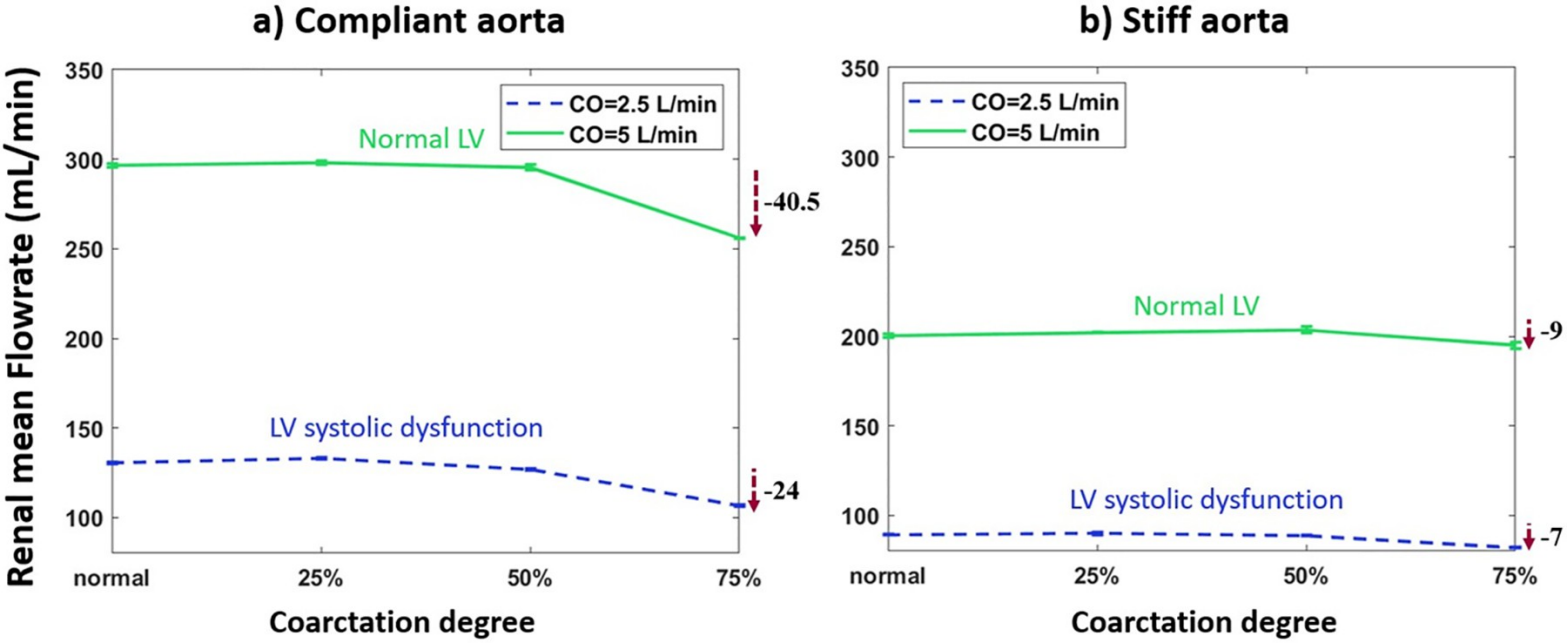
Renal mean flowrate vs. coarctation degree for the a) compliant and b) stiff aortas (PWV = 8 and 28 m/s; AC = 1.2 and 0.35 mL/mmHg) (HR = 75 bpm).

Fig 13 demonstrates renal mean volumetric flowrate vs. coarctation degree for the aortas with highest and lowest compliances (PWV = 8 and 28 m/s; AC = 1.2 and 0.35 mL/mmHg).

#### Variations in kidney pulsatile power transmission with coarctation severity for different aortic compliances

**Fig 14:**
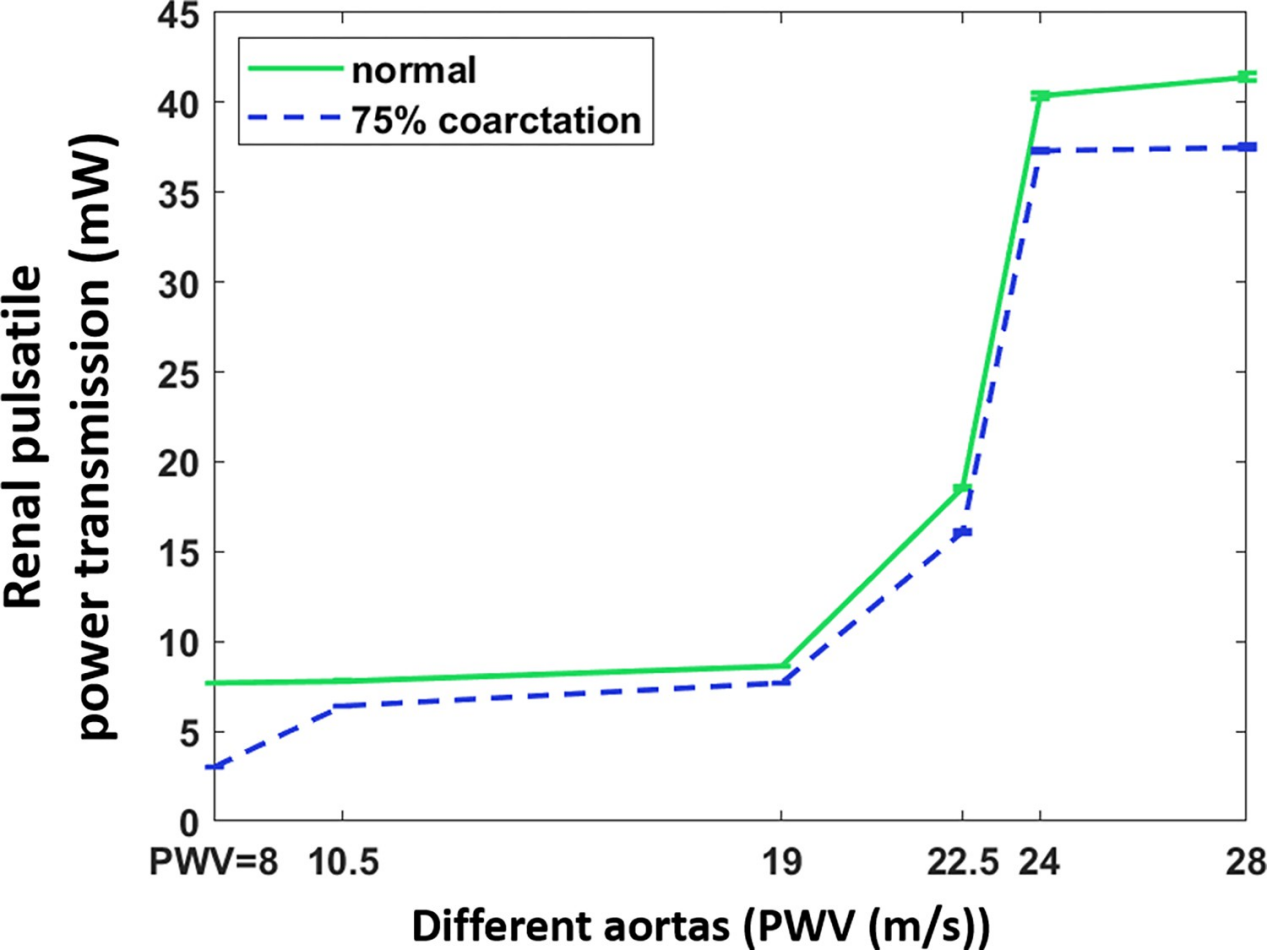
Renal pulsatile power vs. different aortas for normal and 75% coarctation (CO = 5 L/min, HR = 75 bpm).

Fig 14 shows the renal pulsatile power vs. different aortic rigidities for normal condition and 75% coarctation. As the aorta stiffens (due to aging or diseases), renal pulsatile power increases in both normal and CoA cases. The addition of coarctation increases the harmful pulsatile power transmission, but the overall pattern of pulsatile transmission and aortic stiffness remains the same.

The figure showing the energy transmission to the kidneys vs. coarctation degree for different aortic compliances is also provided in the S1 File.

#### The effect of heart rate on energy transmission to the kidneys for normal and coarctation cases

**Fig 15:**
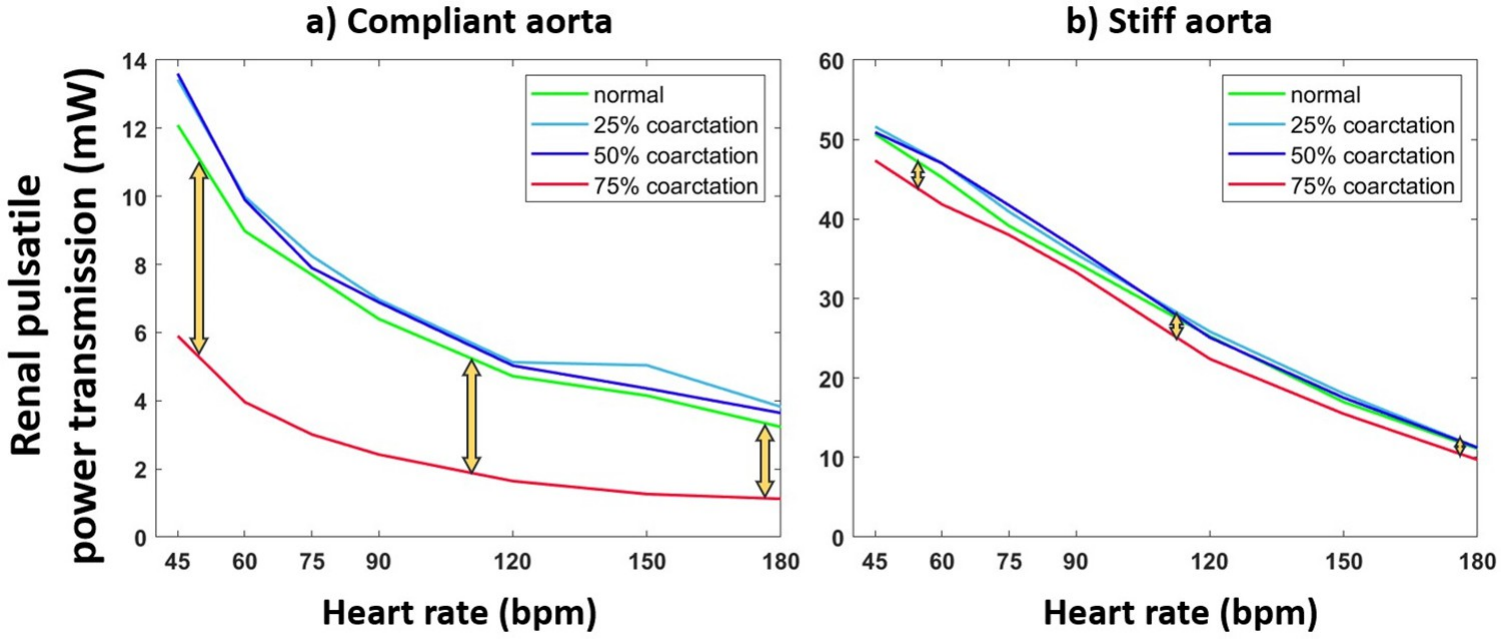
Renal pulsatile power vs. heart rate for a) the most compliant and b) the stiffest aortas (PWV = 8 and 28 m/s; AC = 1.2 and 0.35 mL/mmHg) under normal and coarctation conditions (CO = 5 L/min, HR = 75 bpm). Note that the Renal pulsatile power transmission range for the compliant aorta is 0–14 mW, and for the stiff aorta is 0–60 mW.

Fig 15 demonstrates the Energy transmission to the kidneys as heart rate changes for normal and coarctation cases for the aortas with highest and lowest compliances (PWV = 8 and 28 m/s; AC = 1.2 and 0.35 mL/mmHg). In our setting, the general trend shows a reduction in energy transmission to the kidneys as the heart rate increases, both for normal and coarctation conditions. Yellow arrows demonstrate the difference in renal pulsatile power between normal and 75% coarctation.

#### Wave intensity analysis

**Fig 16:**
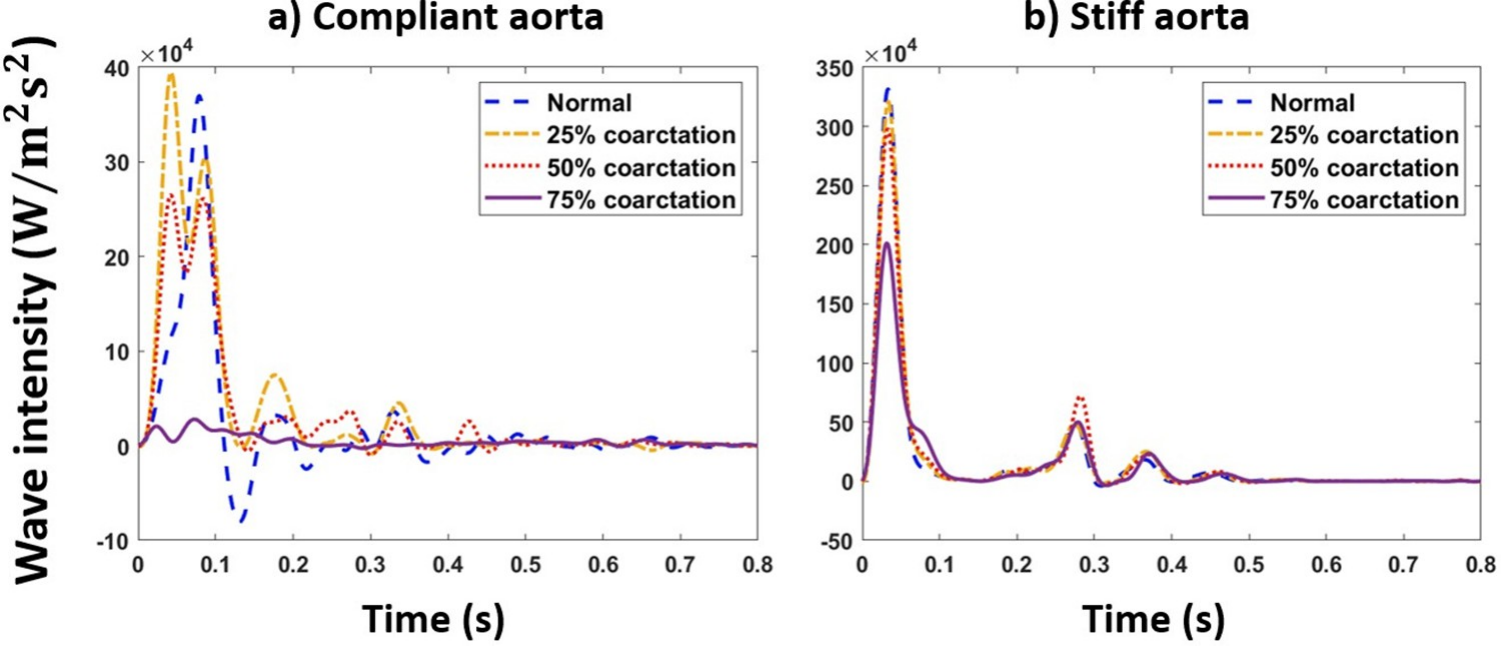
Wave intensity profiles for normal and different coarctation degrees at the renal artery for the a) compliant aorta and b) stiff aorta (PWV = 8 and 28 m/s; AC = 1.2 and 0.35 mL/mmHg), CO = 5 L/min and HR = 75 bpm.

Fig 16 represents the total wave intensity profiles for normal and coarctation cases at the renal artery for the aortas with the highest and lowest compliances (PWV = 8 and 28 m/s; AC = 1.2 and 0.35 mL/mmHg).

**Fig 17:**
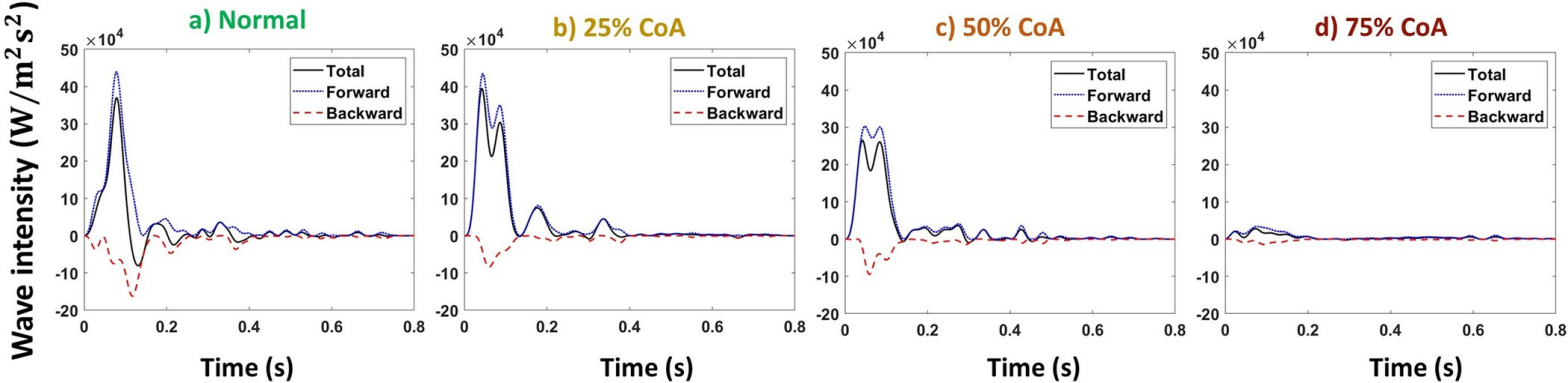
Total, forward, and backward wave intensities at renal artery for the compliant aorta (PWV = 8 m/s; AC = 1.2 mL/mmHg) for a) normal, b) 25%, c) 50%, and d) 75% coarctation.

**Fig 18:**
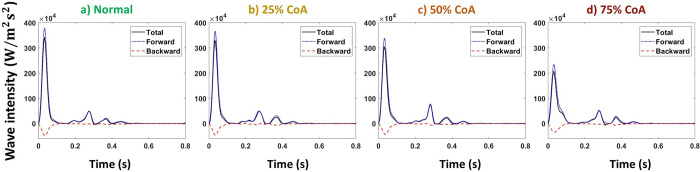
Total, forward, and backward wave intensities at renal artery for the stiff aorta (PWV = 28 m/s; AC = 0.35 mL/mmHg) for a) normal, b) 25%, c) 50%, and d) 75% coarctation.

Figs 17 and 18 represent the total, forward, and backward wave intensity profiles at the renal artery for normal and coarctation cases for the aortas with the highest and lowest compliances (PWV = 8 and 28 m/s; AC = 1.2 and 0.35 mL/mmHg).

#### Power spectrum analysis

**Fig 19:**
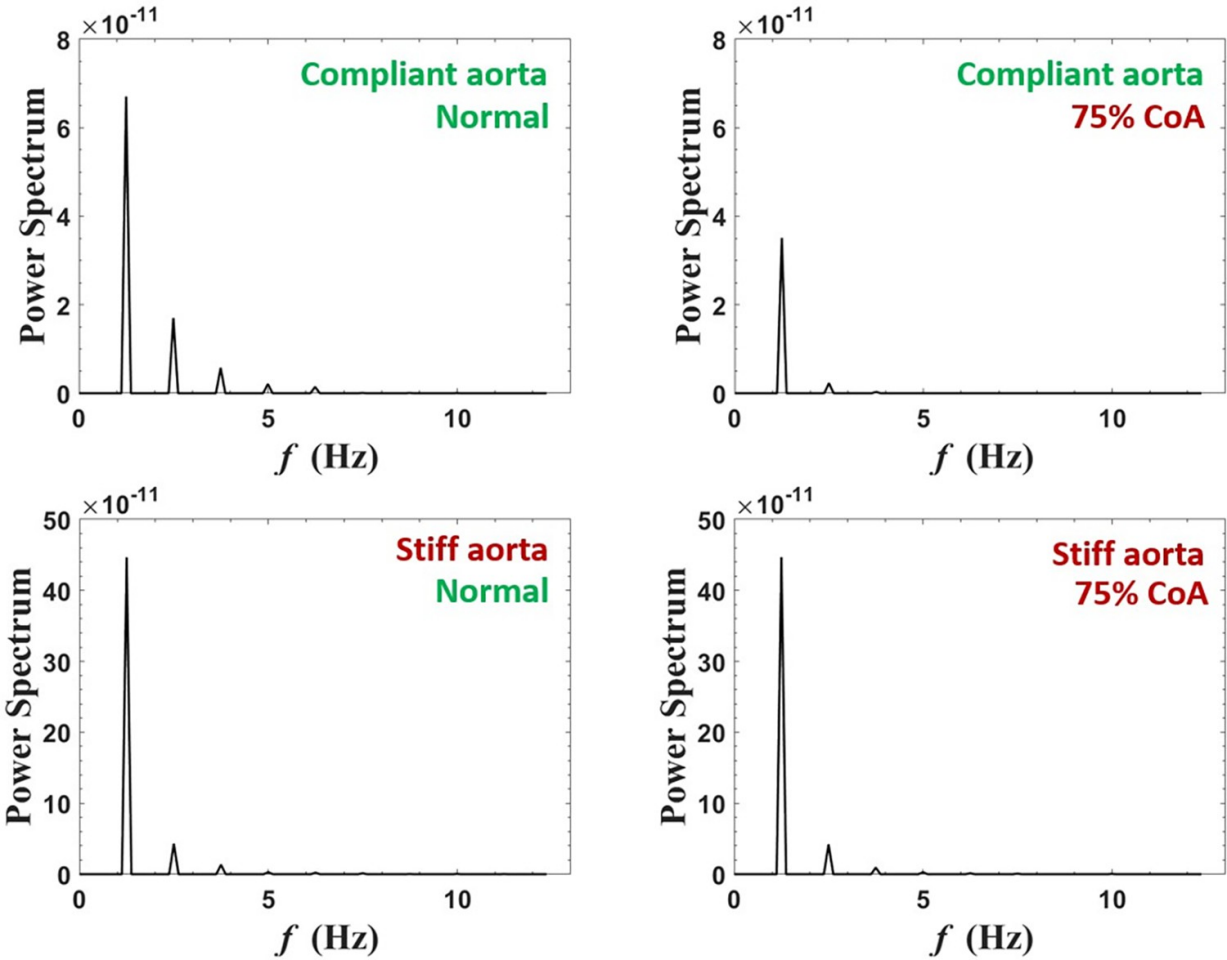
Power spectra of renal artery flow for normal and 75% coarctation cases for the most compliant and the stiffest aortas (PWV = 8 and 28 m/s; AC = 1.2 and 0.35 mL/mmHg) (CO = 5 L/min, HR = 75 bpm).

Power spectrum analysis of blood flow in the renal artery shown in Fig 19 is performed for both normal aorta and 75% CoA condition for the aortas with highest and lowest compliances (PWV = 8 and 28 m/s; AC = 1.2 and 0.35 mL/mmHg). The complete figure, including 25% and 50% coarctation cases, is represented in the S1 File.

## Discussion

In this study, we used a physiologically accurate in-vitro model of the atrioventricular aortic system to investigate the pulsatile hemodynamic mechanisms of aortic coarctation contributing to end-organ damage in the brain and kidneys. Our principal finding is that coarctation of the aorta (CoA) increases cerebral blood flow and harmful pulsatile energy transmission to the brain across all levels of aortic stiffness and CO. Conversely, both renal blood flow and pulsatile energy transmission to the kidneys are reduced in the presence of severe CoA regardless of the level of aortic stiffness and CO. These changes are mainly due to altered wave reflections that occur in CoA. In the following, we provide a comprehensive discussion of the hemodynamic effects of aortic coarctation on the brain and kidneys.

### Aortic coarctation and brain

Previous clinical and preclinical studies [3–6, 38] have shown that CoA impacts wave reflection along the aorta. Our experiments show that the diastolic pressure in the carotid artery almost does not change, while systolic and pulse pressures increase due to the wave reflection, which is consistent with other clinical studies [38]. This shows that our in-vitro model is clinically relevant and physiologically accurate for CoA.

Although the pressure in the carotid artery is an essential factor, recent evidence shows that pulsatile power transmission to the brain may better quantify the potential damage to the brain [39]. In fact, it is the pulsatile feature of the blood that can cause vessel wall growth.

To the best of our knowledge, there is no study quantifying both pressure and flow for investigating pulsatile power transmission to the brain and kidneys in aortic coarctation patients. However, there are studies that have investigated the pulsatile power transmission to the brain in normal aortas. Aghilinejad et al., 2020 [31] reported pulsatile power transmission values to the brain for the normal aortas, which is clinically verified. Their range for aortas without coarctation is between 1–16 mW, and our study shows the same range of the carotid pulsatile power in a normal aorta.

**Fig 20:**
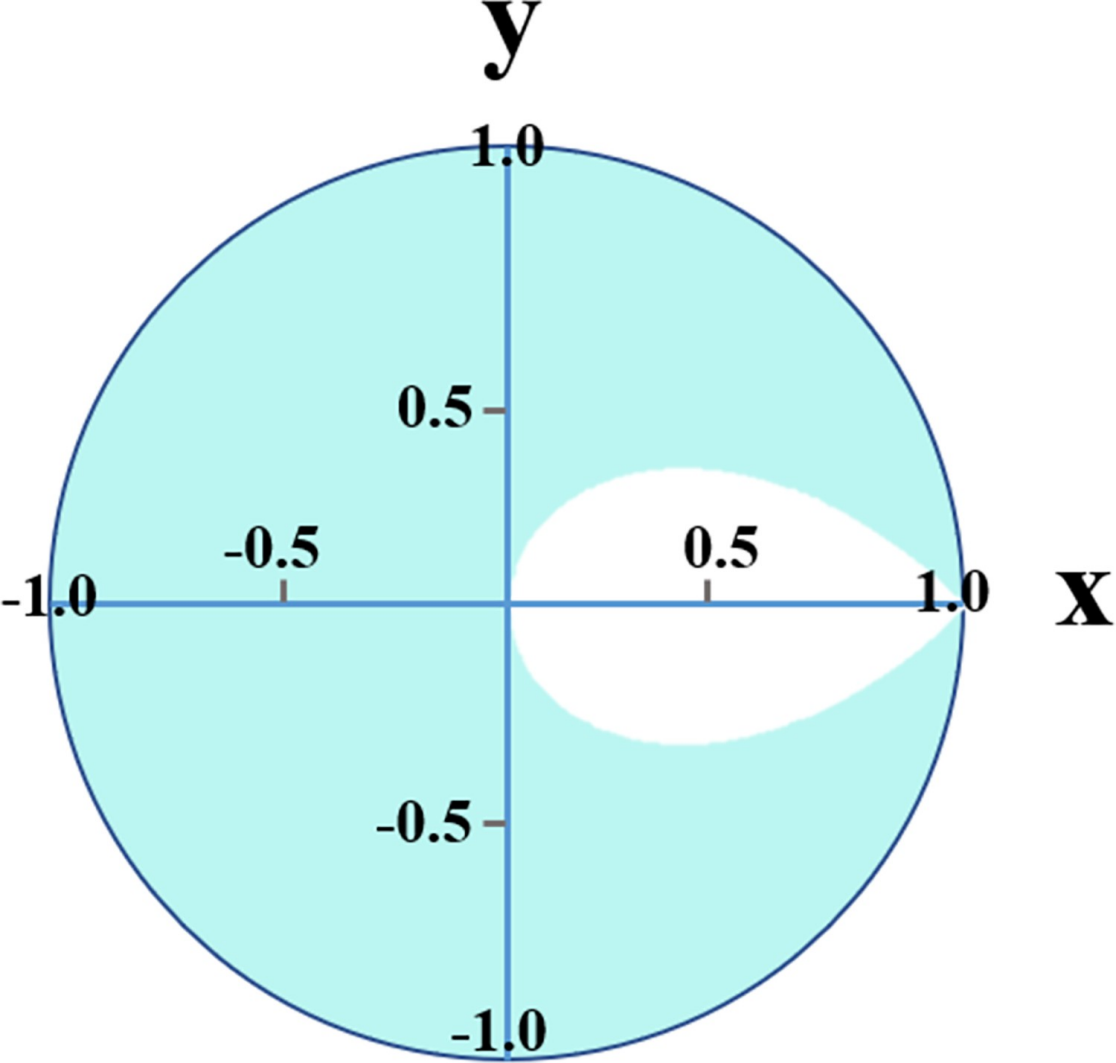
Relationship between the reflection coefficient and pulsatile power (see Pahlevan & Gharib’s 2014 study). $\left(|R|=\sqrt{x^{2}+y^{2}},tan\;\theta =\frac{y}{x}\right)$.

As shown in Fig 4, as the coarctation degree increases, pulsatile power transmission to the brain increases for both compliant and stiffer aortas. This increasing behavior can be explained using the theoretical approximation model proposed by Pahlevan & Gharib [27]. They investigated the effect of wave reflection sites on aortic waves. This model is graphed in a polar plot as shown in Fig 20, where R is associated with the magnitude of reflection and theta is associated with the phase difference between pressure and flow. Note that the figure is in cartesian coordinate where R and theta are related to x and y as $\left(|R|=\sqrt{x^{2}+y^{2}},tan\;\theta =\frac{y}{x}\right)$ The graph shows the relation between the reflection coefficient and pulsatile power. When waves operate in the blue area, the pulsatile power decreases when wave reflection increases. The opposite happens in the white area, where increasing wave reflection increases pulsatile power. In the present study, at the carotid artery, waves operate in the white region, which means that as the reflection coefficient (coarctation degree) increases, flow and pulsatile energy transmission by the waves increase.

As shown in Fig 4, excessive energy transmission to the brain is more considerable after 50% coarctation. Furthermore, this elevation of energy transmission to the brain has a greater magnitude for patients with normal LV than those with LV systolic dysfunction. This happens due to diminished energy propagation in patients with LV systolic dysfunction. Consequently, the impact of the increase in coarctation degree is attenuated. The transmission of excessive pulsatility to the brain is linked to microvascular dysfunction and brain damage, resulting in parenchymal damage and cognitive impairment [39]. As shown in Fig 5, the elevation of the coarctation degree has the same impact on the flow to the brain as it does on the pulsatile energy transmission to the brain.

As demonstrated in Fig 6, results indicate that as the aortic rigidity increases, the amount of pulsatile energy transferred to the brain increases for both normal and coarctation conditions, with a substantial jump after the PWV = 22.5 m/s (age 60–70 years [34]). This indicates the harmful pulsatile energy transmission to the brain is more profound in older patients with coarctation, especially those with diabetes. It should be noted that regardless of the patient’s age, severe aortic coarctation significantly elevates the pulsatile energy transmission to the brain.

At a fixed cardiac output (CO), the transmission of pulsatile energy to the brain decreases with an increasing heart rate. This phenomenon was observed across all levels of aortic rigidity. The addition of aortic coarctation elevates the pulsatile energy at every heart rate. Moreover, severe coarctation (75%) considerably increases pulsatility at all heart rates, while moderate (50%) and mild (25%) coarctation has a much lower impact on pulsatility across all heart rates (ranging from 45 bpm to 180 bpm in our experiments). Notably, the impact of aortic coarctation on pulsatile energy transmission in stiffer aortas—those corresponding to the elderly and patients with diabetes—was insignificant compared to the heart rate effect. For example, a stiff aorta without CoA (represented by the green curve in Fig 7) at a low heart rate of 50 bpm would have higher harmful pulsatile energy transmission to the brain than an aorta with the same stiffness at a higher heart rate (e.g., 80 bpm) but with severe coarctation (represented by the red curve in Fig 7).

Our results show that the first peak of the total wave intensity amplifies as the coarctation degree increases (Fig 8). This amplification is more pronounced beyond 50% coarctation. In normal cases (without CoA), the ratio of the first peak of the forward wave intensity to the backward wave intensity is smaller than that of those with CoA (see Figs 9 and 10). This suggests that additional reflection caused by CoA plays a dominant role in altering wave reflection, resulting in increased forward wave transmission to the brain. This increased forward wave transmission amplifies pulsatile energy transmission and directs a greater volume of flow toward the brain.

The power spectrum analysis of the carotid artery blood flow (Fig 11) shows a fundamental peak at 1.25 Hz and a harmonic at 2.5 Hz. The amplitude of these peaks increases significantly in the presence of CoA, indicating its effect on enhancing blood flow toward the brain.

### Aortic coarctation and kidneys

As most clinical studies have focused on severe coarctation cases, they have reported low renal mean flow rates caused by CoA [40, 41]. As shown in Figs 12 and 13, findings indicate a non-linear behavior in both renal pulsatile power and mean flow rate. Up to a 25% degree of coarctation, almost no change is observed in renal pulsatile power and renal mean flow rate. After that, a reducing trend starts with a pronounced reduction beyond the 50% coarctation. While clinical studies suggest that reduced renal pulsatile energy has a pronounced effect on the kidney and can lead to acute renal insufficiency or failure [23], to the best of our information, no study has reported pulsatile power transmission to the kidneys. And this is the first study focusing on the changes in pulsatile power transmission to the kidneys in aortic coarctation patients.

The mentioned non-linear behavior can again be explained by Pahlevan & Gharib’s analytical approximation [27]. Up to 25% coarctation, the waves operate in the white area, close to the border of the white and the blue region. Therefore, with the increase of coarctation degree, renal pulsatile power slightly increases. After that, the operating point shifts to the blue region, followed by a notable drop in the renal pulsatile power. Furthermore, the drop in pulsatile energy transmission to the kidneys is more considerable for patients with normal LV compared to patients with LV systolic dysfunction. This can be explained based on the conservation of energy. In normal LV condition, as pulsatile energy to the brain increases with the coarctation degree increase, less amount of energy goes to the kidneys. Results show that the effect of the coarctation degree increase on flow to the kidneys follows the same trend as the pulsatile energy transmission to the kidneys.

Results presented in Fig 14 indicate that as the aorta gets more rigid, the amount of energy transferred to the kidneys increases for both normal and coarctation conditions, with a substantial jump after the PWV = 22.5 m/s (age 60–70 years [34]). This suggests the harmful pulsatile energy transmission to the kidneys is more profound in older patients with coarctation, especially those with diabetes. It should be noted that regardless of the patient’s age, severe aortic coarctation decreases the energy transmission to the kidneys.

The presence of CoA does not negatively impact the transmission of pulsatile energy to the kidneys, as most of the wave energy is diminished by the presence of the CoA upstream of the renal arteries. Similar to the carotid artery, pulsatile energy transmission in the renal artery decreases with the increase in heart rate at a fixed CO and across all aortic rigidities (Fig 15). However, the addition of aortic coarctation reduces the pulsatile energy transmission in the renal arteries at every heart rate, with a more profound relative effect for severe coarctation (75%) in compliant aortas (younger patients).

As shown in Fig 16, our results show that up to 25% coarctation, the first peak of the total wave intensity almost does not change. After that, the first peak decreases, which is more pronounced beyond 50% coarctation. The behavior of wave intensity at the renal artery for different degrees of CoA explains the non-linear behavior of renal pulsatile power transmission and blood flow observed in the results. As presented in Figs 17 and 18, in normal cases (without CoA), the ratio of the first peak of the forward wave intensity to the backward wave intensity is larger than those with CoA. This indicates the contribution of extra reflection (CoA) in altering wave reflection, resulting in decreased forward wave transmission to the kidneys.

As shown in Fig 19, power spectrum analysis for blood flow at the renal artery shows a fundamental peak at 1.25 Hz followed by the other harmonics that are easily discerned. The figure demonstrates that severe CoA dampens flow harmonics. This explains the reduced blood flow to the kidneys in the presence of severe coarctation. In this context, our study’s findings indicate that the reduction of blood flow to the kidneys is not solely due to the downstream resistance caused by CoA but also to the wave interactions. While CoA introduces resistance to flow, as exemplified by a CoA severity of 75%, an analysis focusing solely on the resistance effect -according to the Poiseuille equation ($F\propto \frac{r^{4}\Delta P}{ȠL}$)-, predicts a reduction in downstream flow to 1/16 of the baseline. This means that renal volume blood flow would have been impacted to the same magnitude if the driving mechanism was simply the resistance and not the complex wave phenomenon. In fact, as evidenced by our results (see Fig 13), the alteration in flow caused by CoA is significantly less pronounced than anticipated by the resistance-based Poiseuille equation. This observation holds true for the augmentation of carotid blood flow as well. Power spectrum analysis also suggests that the effect of extra reflection (coarctation) is more prominent in the compliant aorta (younger patients) rather than the stiff aorta (older patients), as the harmonics are dampened more significantly. In the compliant aorta, severe coarctation makes the third harmonics onwards almost disappear.

### Limitations

Our in-vitro atrioventricular-aortic simulator includes all three branches at the aortic arch, coronary arteries, renal arteries, and iliac bifurcation, but it does not include the other small branches in the descending to abdominal section of the aorta. Since these branches create minimal wave reflection and have a negligible effect on the global pulsatile hemodynamics of the aorta, this exclusion does not affect the overall findings and conclusions of this study.

Given the complexities of the circulatory system, the dynamic adjustments in end-organ resistance and compliance regulated by neurologic systems are not included in this study. Future research could explore these effects in detail using a combination of numerical methods and simulation tools.

## Conclusion

Our results suggest that CoA does not impair the blood flow to the brain. However, it increases pulsatile energy transmission to the brain, which is particularly evident after 50% coarctation. This can promote neurodegenerative diseases such as dementia [39] and may accelerate the rupture of brain aneurysms if present [42].

In our study, CoA has a non-linear effect on both renal blood flow and pulsatile power transmission to the kidneys. As the coarctation degree increases, there is almost no change in pulsatile power transmission and blood flow to the kidneys up to 50% coarctation. However, renal blood flow significantly decreases in cases with severe COA (>50%). Reduced blood flow to the kidneys can potentially result in renal impairment [23]. The pulsatile power transmission to the kidneys is also reduced at severe COA.

The findings of this study contribute to an understanding of the underlying hemodynamic mechanisms of CoA and its impact on end-organ damage. This is an important step toward developing improved therapeutic strategies for CoA patients. The insight from our study highlights the importance of considering age-related factors, coarctation severity, patient’s cardiac function (e.g., cardiac output), and baseline heart rate when evaluating the altered hemodynamics at the end organs in COA patients.

## Supporting information

S1 File (DOCX)
