# Supplementary material for: The global effect of aortic coarctation on carotid and renal pulsatile hemodynamics
Source: PLoS One. 2024 Dec 17;19(12):e0310793. doi: 10.1371/journal.pone.0310793 (PMC11651599; doi:10.1371/journal.pone.0310793)
Supplement: S1 File — (DOCX) [file pone.0310793.s001.docx]

**Supplementary materials**

**Artificial organs fabrication**

The artificial organs used in the system (aorta, LV, and LA) are fabricated using natural latex rubber (Chemionics Corp.) and silicone rubber (RTV-3040, Freeman Manufacturing & Supply Company). These materials are selected because they have the characteristic properties of mimicking the stiffness of a natural aorta (1). The artificial aortas are created using molds that are scaled to match the size of a human aorta, including the ascending aorta, aortic arch, thoracic aorta, abdominal aorta, and major branches. The fabrication process involves the following steps: For latex aorta: First, the stainless-steel metal aortic mold is dipped into a container filled with liquid latex; then, the mold is removed from the container, followed by curing the coated material at standard room temperature (25˚C) for two hours. If necessary, additional coating layers were applied to achieve the desired aortic compliance. For silicone aorta: At first, the base of the silicon rubber (RTV-3040, Freeman Manufacturing & Supply Company) and the catalyst were mixed with a mass ratio of 10 (base) to 1 (catalyst); then, by using a soft-tip acrylic brush, we coated a light silicon sheet of 25 g of the mixed solution. We let the coated material for 16 hours at standard room temperature (25˚C); finally, we repeated the coating for more layers (as required) to achieve the desired aortic compliance. Figure 1 and table 1 represent the segments and related dimensions of the artificial aorta. LV and LA were fabricated using a procedure similar to that of silicone-based aortas. Using a 3D printer, the designed molds were printed for LV and LA; then, the above-mentioned fabrication process was done.

| **Segment name** | **Length (mm)** | **Inlet diameter (mm)** |
| --- | --- | --- |
| **1.**Ascending Aorta | 70 | 24 |
| **2.**Aortic Arch | 60 | 18 |
| **3.**Thoracic Aorta | 170 | 20 |
| **4.**Renal Artery | 65 | 5 |
| **5.**Abdominal Aorta | 130 | 16 |
| **6.**Iliac Artery | 76 | 12 |
| **7.**Coronary Artery | 45 | 4 |
| **8.** Brachiocephalic Artery | 34 | 12.4 |
| **9.**Left Common Carotid Artery | 70 | 8.1 |
| **10.**Left Subclavian Artery | 100 | 8.4 |


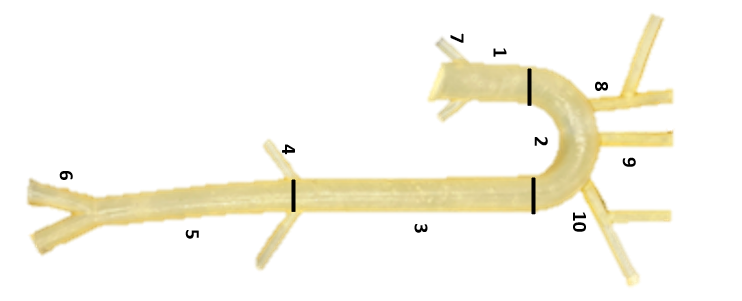


Figure 1: Segmented schematic of the artificial aorta

Table 1: geometric properties of the aorta mold

| **PWV (m/s)** | **10.5** | | | | **19** | | | | **22.5** | | | | **24** | | | |
| --- | --- | --- | --- | --- | --- | --- | --- | --- | --- | --- | --- | --- | --- | --- | --- | --- |
|  | **normal** | **25%** | **50%** | **75%** | **normal** | **25%** | **50%** | **75%** | **normal** | **25%** | **50%** | **75%** | **normal** | **25%** | **50%** | **75%** |
| **Carotid pulsatile power transmission (Normal LV) (mW)** | 6.8 | 8.6 | 9 | 13.1 | 11.9 | 12.9 | 13 | 22.5 | 11.5 | 12.6 | 12.7 | 16 | 39.4 | 41.3 | 42.4 | 44.6 |
| **Carotid pulsatile power transmission (LV systolic dysfunction)**  **(mW)** | 3.81 | 4.48 | 4.71 | 5.56 | 5 | 5.3 | 5.5 | 7.6 | 5.2 | 5.7 | 5.8 | 6.4 | 14.3 | 14.4 | 14.6 | 15 |

**The effect of coarctation degree on pulsatile power transmission to the brain**

Table 2: Carotid pulsatile power transmission in normal LV and LV systolic dysfunction for different aortic compliances, for normal and different coarctation degrees.

**Variations in brain pulsatile power transmission with coarctation severity for different aortic compliances**

Figure 2 shows the Carotid pulsatile power vs. coarctation degree for six different aortic compliances.


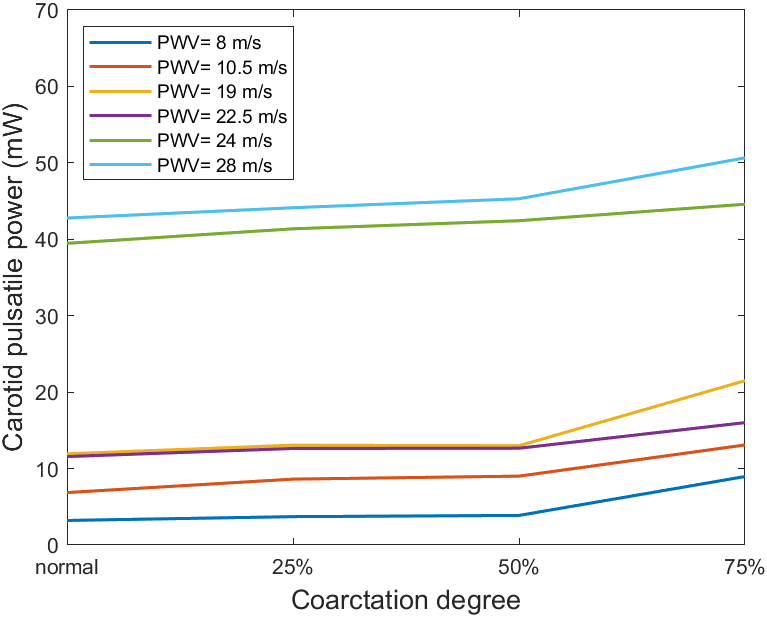


Figure 2: Carotid pulsatile power vs. coarctation degree for different aortic compliances (CO=5 L/min, HR= 75bpm).

**Power spectrum analysis of blood flow at the carotid artery**


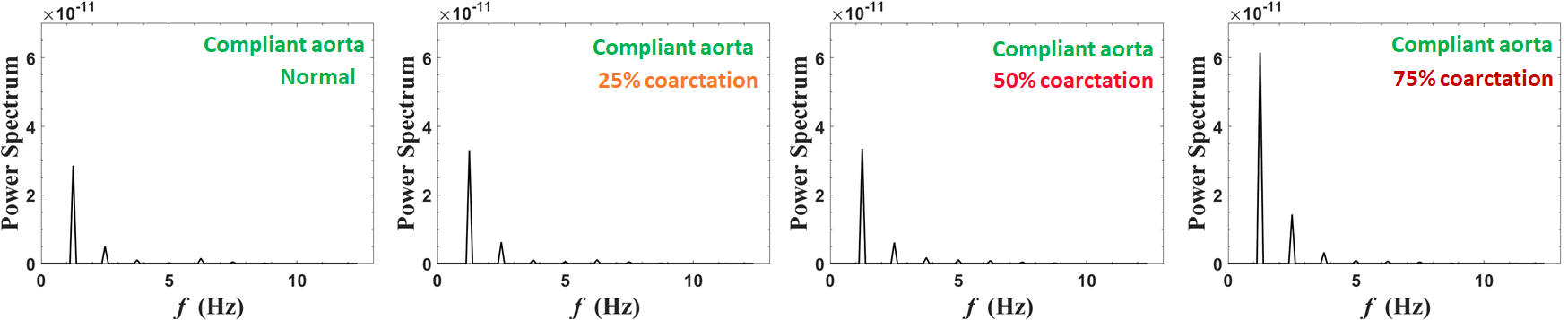

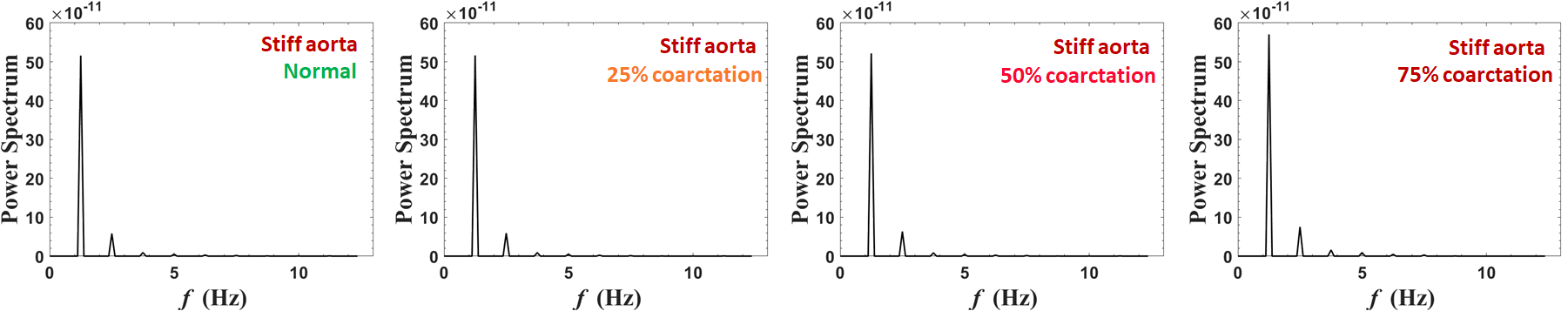


Figure 3: Power spectra of carotid artery flow for normal, 25%, 50%, and 75% coarctation cases for the most compliant and the stiffest aortas (PWV=8 and 28 m/s; AC= 1.2 and 0.35 mL/mmHg) (CO= 5 L/min, HR= 75 bpm)

**The effect of coarctation degree on pulsatile power transmission to the kidneys**

| **PWV (m/s)** | **10.5** | | | | **19** | | | | **22.5** | | | | **24** | | | |
| --- | --- | --- | --- | --- | --- | --- | --- | --- | --- | --- | --- | --- | --- | --- | --- | --- |
|  | **normal** | **25%** | **50%** | **75%** | **normal** | **25%** | **50%** | **75%** | **normal** | **25%** | **50%** | **75%** | **normal** | **25%** | **50%** | **75%** |
| **Renal pulsatile power transmission (Normal LV) (mW)** | 7.6 | 8.9 | 8.5 | 6.4 | 6.6 | 8.5 | 8.7 | 6.2 | 18.5 | 20.2 | 19.8 | 16.1 | 39.47 | 40 | 39.9 | 37.4 |
| **Renal pulsatile power transmission (LV systolic dysfunction)**  **(mW)** | 3.30 | 3.87 | 3.85 | 3.30 | 2.1 | 2.8 | 2.9 | 2.5 | 6.7 | 7.3 | 7.4 | 6.2 | 14.4 | 15 | 14.9 | 12.8 |

Table 3: Renal pulsatile power transmission in normal LV and LV systolic dysfunction for different aortic compliances, for normal and different coarctation degrees.

**Variations in kidney pulsatile power transmission with coarctation severity for different aortic compliances**

**
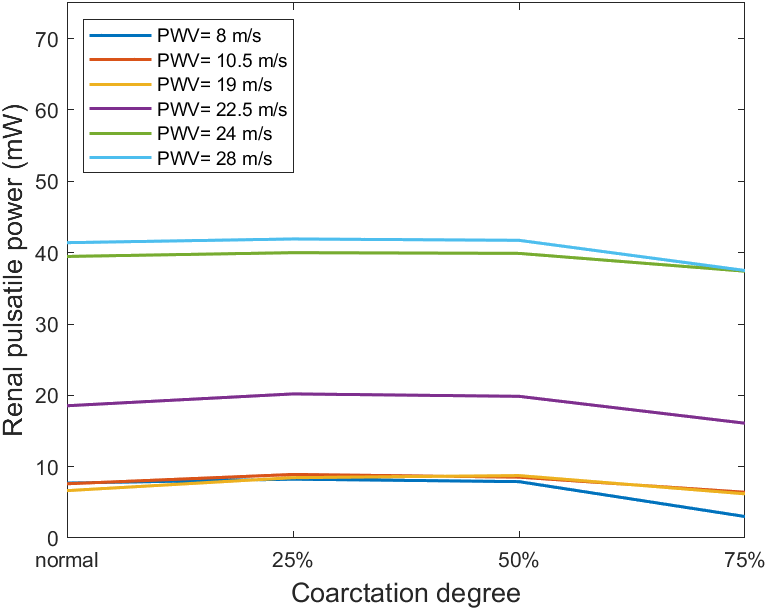
**

Figure 4: Renal pulsatile power vs. coarctation degree for different PWVs (CO=5 L/min, HR=75 bpm).

**
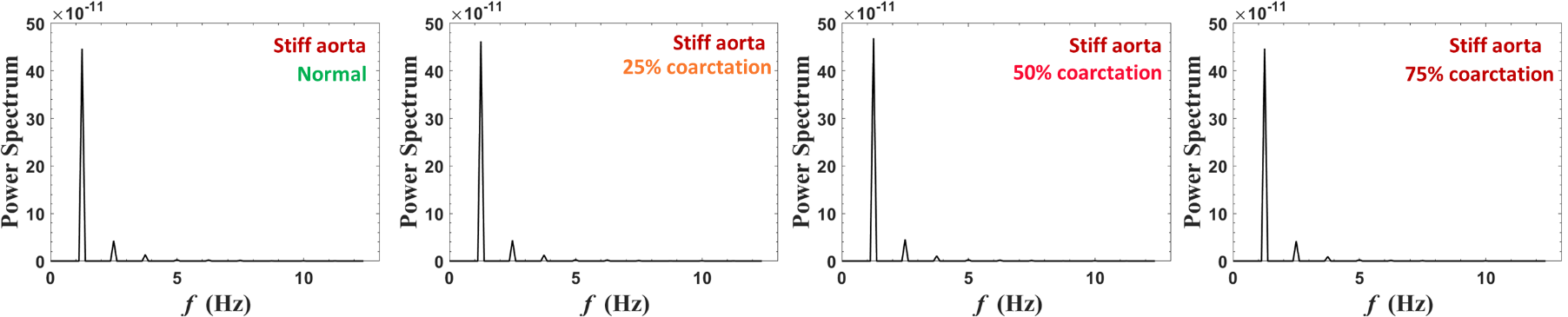
**
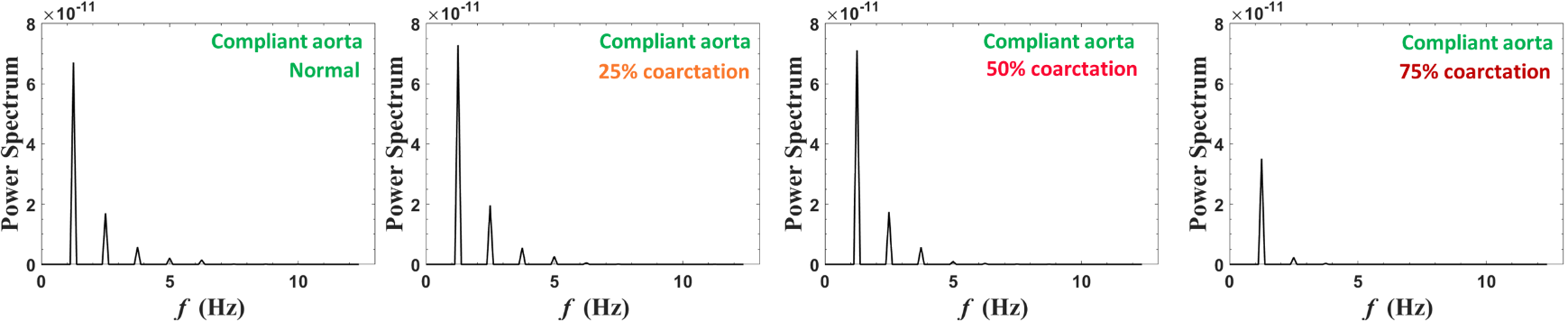
**Power spectrum analysis of blood flow at the renal artery**

Figure 5: Power spectra of renal artery flow for normal, 25%, 50%, and 75% coarctation cases for the most compliant and the stiffest aortas (PWV=8 and 28 m/s; AC= 1.2 and 0.35 mL/mmHg) (CO= 5 L/min, HR= 75 bpm)

**References:**

1. Pahlevan NM, Gharib M. A bio-inspired approach for the reduction of left ventricular workload. PloS one. 2014;9(1):e87122.
